# Metabolically diverse microorganisms mediating hydrocarbon cycling in the subseafloor sediment of the Challenger Deep

**DOI:** 10.1128/mbio.03943-25

**Published:** 2026-04-13

**Authors:** Xinxin He, Jiwen Liu, Haojin Cheng, Xiaoyu Zhu, Heyu Lin, Da-Wei Li, Yizi Yang, Ronghua Liu, Derui Song, Yanfen Zheng, David J. Lea‑Smith, Nikolai Pedentchouk, Jonathan D. Todd, Meixun Zhao, Xiao-Hua Zhang

**Affiliations:** 1 Frontiers Science Center for Deep Ocean Multispheres and Earth System, College of Marine Life Sciences, Ocean University of China 12591, Qingdao, China; 2 Laboratory for Marine Ecology and Environmental Science, Qingdao Marine Science and Technology Center 554912 , Qingdao, China; 3 College of Marine Sciences, Fujian Agriculture and Forestry University 12449, Fuzhou, China; 4 Key Laboratory of Evolution & Marine Biodiversity (Ministry of Education), Institute of Evolution & Marine Biodiversity, Ocean University of China 12591, Qingdao, China; 5 School of Biological Sciences, University of East Anglia, Norwich, United Kingdom; 6 School of Geography, Earth and Atmospheric Sciences, The University of Melbourne, Parkville, Victoria, Australia; 7 Key Laboratory of Marine Chemistry Theory and Technology, Ministry of Education, Ocean University of China 12591, Qingdao, China; 8 Department of Geosciences, College of Petroleum Engineering and Geosciences, King Fahd University of Petroleum & Minerals 48080, Dhahran, Saudi Arabia; 9 Laoshan Laboratory 474988, Qingdao, China; Georgia Institute of Technology, Atlanta, Georgia, USA

**Keywords:** Challenger Deep, Mariana Trench, hadal sediment, microbial communities, hydrocarbon metabolism

## Abstract

Hadal subseafloor sediments host abundant and active microbial biosphere with considerable heterotrophic activity. However, carbon and nutrient cycling processes and mechanisms driven by hadal subsurface microorganisms remain poorly understood. Using culture-dependent and culture-independent methods, we characterized the diversity, metabolism, and vertical dynamics of hydrocarbon-degrading (HYD) bacteria in a subsurface sediment core (MT20-750, ~750 cm below seafloor [cmbsf]) collected from the Challenger Deep (10,816 m below sea level) in the Mariana Trench. The sediment core contained high concentrations of mid- and long-chain *n*-alkanes (310–8,724 ng/g), although no <C_18_ *n*-alkanes at detectable levels were present, in contrast to proximal hadal seawater samples where these compounds were highly abundant. Metagenomic analysis identified diverse genes for aerobic and anaerobic degradation of *n*-alkanes and aromatic compounds, distributed across a wide range of taxa, dominated by Chloroflexota, Planctomycetes, Proteobacteria, and Actinobacteria, alongside six novel HYD phyla. Metabolic reconstruction of 120 HYD metagenome-assembled genomes (MAGs) suggests distinct hydrocarbon degradation preferences and metabolic strategies among different bacterial groups. Additionally, hadal bacterial isolates from Proteobacteria, Actinobacteria, and Firmicutes were able to degrade *n*-alkanes (C_18–36_), with *Dietzia maris* HXX048 removing ~50% of *n*-eicosane within 45 days under 5°C and 50 MPa. Predicted substrate binding of C_10_ and C_20_ with AhyA and heterologous expression of *almA* in Chloroflexota genomes supported their degradative capacity under anaerobic and aerobic conditions. The detection of putative hydrocarbon synthesis genes, specifically *oleBC* and *oleC*, suggests that hadal heterotrophic microorganisms may synthesize hydrocarbons. These findings provide evidence for microbial hydrocarbon production and thereby support a previously unrecognized sedimentary hydrocarbon cycle.

**IMPORTANCE:**

Our findings suggest that hydrocarbon degradation may play an important role in organic matter decomposition and carbon cycling in the hadal subseafloor. This degradation capacity is likely distributed through diverse metabolic pathways across a wide range of phylogenetic taxa. The detection of genes likely encoding enzymes involved in aerobic and anaerobic hydrocarbon degradation, as well as the identification of novel hydrocarbon-degrading (HYD) phyla, highlights the complexity and significance of microbial processes in hadal subsurface sediment. The widespread distribution of hydrocarbon degradation capacity in different hadal sediments suggests hydrocarbons as a potential carbon source sustaining microbial life in this extreme environment. Moreover, the presence of genes associated with hydrocarbon synthesis suggests that hadal sediment microbes possess the genetic potential for both degrading and producing hydrocarbons, pointing to a dynamic and multifaceted hydrocarbon cycle within hadal subsurface sediment.

## INTRODUCTION

The hadal zone, encompassing ocean depths below 6,000 m, primarily consists of deep trenches and ranks among Earth’s least-explored aquatic biospheres (1–3). The sedimentary ecosystem represents the largest and most distinctive habitat in the hadal realm, characterized by unique biogeochemical characteristics (2–6). The extreme and special habitat conditions of hadal sediments have fostered a distinct microbial ecological landscape characterized by remarkable novelty, as highlighted by a recent study showing that ~89.4% of species-level metagenome-assembled genomes (MAGs) were previously unrecognized (7). The unique “V-shaped” geomorphology and specific deposition dynamics of hadal trenches also lead to the concentration of organic matter in the sediment (8–11). This concentrated organic matter supports elevated rates of carbon turnover and sustains diverse chemoheterotrophic microbial communities (7, 9). Moreover, the extreme environment in the hadal sediment has shaped unique microbial communities with distinct metabolic potentials, including the degradation of persistent organic pollutants such as certain refractory organic matter, polychlorinated biphenyls (PCBs) (12), and hydrocarbons (4) and transformation or removal of heavy metals (13, 14).

Hydrocarbons are widely distributed in the ocean, primarily originating from natural oil seeps, human activities, and biological production by cyanobacteria, algae, and higher terrestrial plants (15–17). Short- to mid-chain *n*-alkanes (C_5–24_) are rapidly degraded by colocalized hydrocarbon-degrading (HYD) microorganisms, although some likely accumulate and settle into the deeper water column (15–17). Other hydrocarbons, including mid- and long-chain *n*-alkanes and polycyclic aromatic hydrocarbons (PAHs), accumulate in the deep ocean (12, 15). Recent studies have reported *n*-alkane concentrations in Mariana Trench sediments at depths of 4,900–7,068 m, ranging from 0.34 to 3.47 μg/g dry weight (dw) (18). Additionally, abundant *n*-alkanes (average: 23.5 μg/g dw) have also been observed at 2,000–6,000 m water depths and in the hadal surface sediments (<10 cm below seafloor [cmbsf]) at approximately 10,900 m (average: 2.3 μg/g dw) (4). These hydrocarbons likely serve as key carbon sources in the hadal ecosystem.

Previous studies reported abundant HYD microorganisms in the deep water column (>6,000 m) of the Mariana Trench (4, 19), particularly in the near-bottom layers at 10,400 and 10,500 m (4). Dominant genera included *Oleibacter*, *Thalassolituus,* and *Alcanivorax*, all of which comprise aerobic species capable of metabolizing aliphatic hydrocarbons (4). As significant sinks for organic matter, the hadal sediments accumulate hydrocarbons through both gradual settling and episodic deposition of slope-derived material triggered by frequent seismic events (4, 20). The latter process leads to discontinuous sedimentation and supplies additional hydrocarbons for hadal subseafloor microbial communities. Although HYD microbiomes have been identified in hadal sediments (13, 21, 22), their diversity, vertical distribution, and metabolic characteristics remain poorly understood. This study aimed to investigate whether hadal sediments host abundant yet distinct populations of HYD bacteria compared with those in the water column and to determine their potential for hydrocarbon degradation. Additionally, based on our earlier carbon and hydrogen stable isotope evidence that unknown heterotrophic organisms contribute to hydrocarbon production in surface sediments of the hadal zone (4), a further objective was to explore whether *in situ* hydrocarbon consumption and production occur simultaneously, potentially facilitating a previously unrecognized hydrocarbon cycle in this extreme environment.

To address these objectives, we conducted a comprehensive analysis of HYD microorganisms and their metabolic strategies in a hadal sediment core from the Challenger Deep (water depth: 10,875 m, core length: ~7.5 m) (Fig. S1). Combining geochemical detection of *n*-alkane concentrations, 16S rRNA gene amplicon and metagenome sequencing, enrichment cultures, and physiological characterization, we identified concentrations of *n*-alkanes (C_18_–C_32_) in the hadal subsurface sediments and observed a significant positive correlation between the abundance of *n*-alkane degradation genes and *n*-alkane concentrations across sediment layers. Compared to the hadal seawater, metagenomic analysis revealed distinct HYD communities in hadal sediments, with Chloroflexota representing the most abundant clade. Culturable isolates from Actinobacteria, Proteobacteria, and Firmicutes were experimentally verified for their HYD capabilities, and HYD genes from Chloroflexota were heterologously expressed to confirm their ability to degrade hydrocarbons. Furthermore, using publicly available metagenomic and metatranscriptomic data, we demonstrated the active expression and consistent presence of HYD microbes across different hadal sediments. Overall, our study demonstrates that diverse HYD microorganisms have the potential to participate in hydrocarbon cycling in the deep-sea environment of the Challenger Deep.

## RESULTS

### *n*-Alkane concentrations in the sediment core

**Fig 1:**
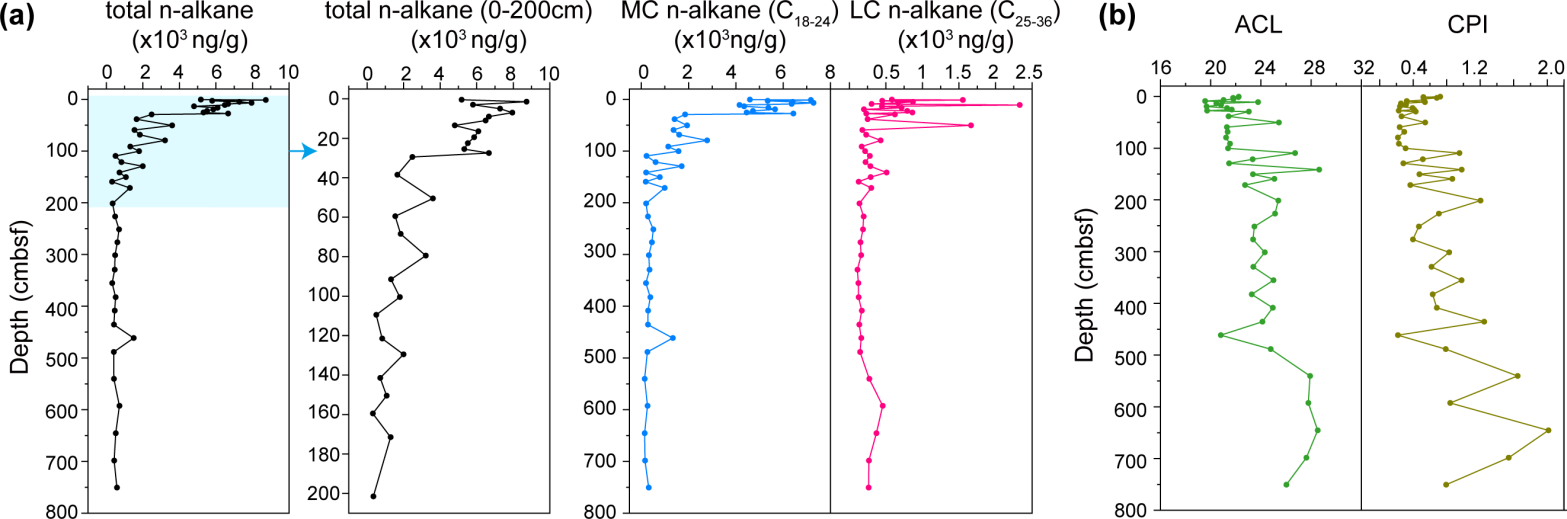
The *n*-alkane content and the average chain length (ACL) and carbon preference index (CPI) values of *n*-alkane in the hadal sediment core. (**a**) Total *n*-alkane, mid-chain (C_18–24_), and long-chain (C_25–36_) *n*-alkane content in the sediment core. (**b**) The ACL and CPI values of *n*-alkanes in the hadal sediment core. ACL: average chain length; CPI: carbon preference index.

We first quantified *n*-alkane concentrations across 45 subsamples. Amounts varied from 310 ng/g (158–161 cmbsf) to 8,724 ng/g (1–2 cmbsf), accounting for approximately 0.01%–0.12% of the total organic carbon (TOC; Table S1) (2). Mid-chain *n*-alkanes (C_18–24_, 27.8%–96.6%) were more abundant than long-chain *n*-alkanes (C_25–36_, 3.4%–72.2%), while short-chain *n*-alkanes (<C_18_) were not detected (Fig. 1a; File S2). The total *n*-alkane concentration was highest in the top 30 cmbsf (2,475–8,724 ng/g), rapidly decreasing with depths until 200 cmbsf (Fig. 1a). Below 200 cmbsf, total *n*-alkane concentrations remained relatively stable but at a lower range of 321–1,493 ng/g, with an average of 559 ng/g.

To characterize the sources of *n*-alkanes, we analyzed two indices: average chain length (ACL) and carbon preference index (CPI) (Fig. 1b). The ACL values ranged from 19.6 to 28.6 and gradually increased with depth, with some discrete exceptions at 100, 140, and 540 cmbsf. CPI values for total *n*-alkanes fluctuated between 0.22 and 2.01 (average: 0.63) from 0 to 200 cmbsf <1 (Fig. 1b; Fig. S2 and Table S1). The dominant mid-chain *n*-alkanes exhibited a predominance of even-chain carbon (Fig. S2, Table S1), which is indicative of biosynthesis by heterotrophic bacteria (4).

### Diverse hydrocarbon degradation genes in the hadal subseafloor

**Fig 2:**
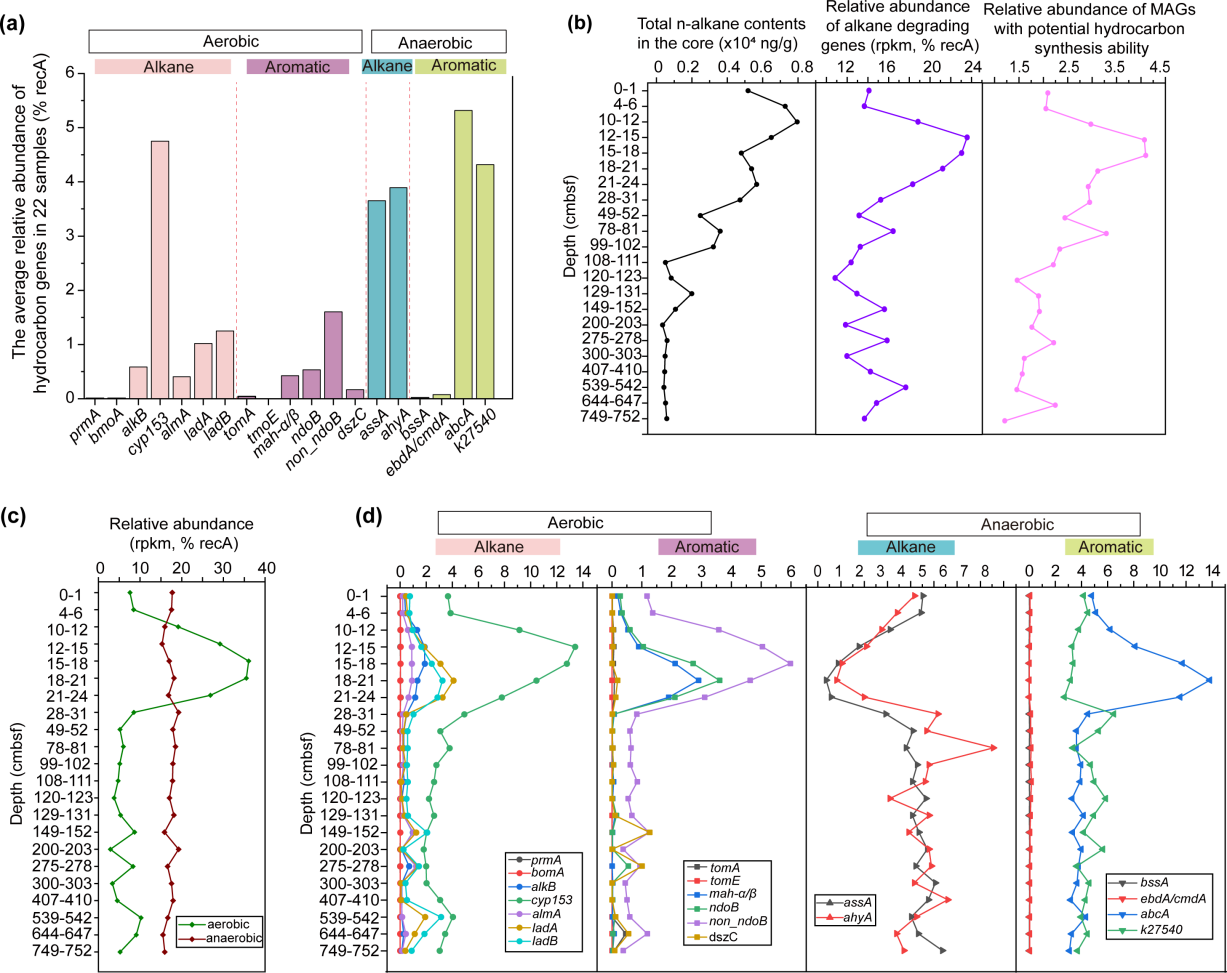
Diverse hydrocarbon degradation genes in the core. (**a**) Variation trends of the relative abundance of each HYD gene group across the sediment core. (**b**) Depth distributions of *n*-alkane content, *n*-alkane degradation genes, and hydrocarbon synthesis MAGs. (**c**) Relative abundance of genes for aerobic and anaerobic hydrocarbon degradation with sedimentation depth. (**d**) Relative abundance of genes involved in aerobic (alkane and aromatic) and anaerobic (alkane and aromatic) degradation across the sediment core.

To explore the genetic basis of hydrocarbon degradation, we investigated the abundance of HYD genes in metagenomes across 22 different sediment depths. A total of 2,400 genes related to hydrocarbon degradation were detected (Fig. 2; Tables S4 and S5). The relative abundance of putative alkane-degrading genes was significantly higher than that of putative aromatic-degrading genes (16.48% vs 12.33%, *P*-value < 0.01, paired *t*-test based on CLR-transformed data) (Table S5). Among genes potentially involved in aerobic alkane oxidation, those encoding alkane hydroxylase (*cyp153*, targeting C_5–16_) were the most abundant, followed by *ladAB* (targeting C_15–36_), *almA* (targeting C_20–32_), and *alkB* (targeting C_12–26_) (Fig. 2a). These genes exhibited a higher relative abundance in the upper sediment layers (0–30 cmbsf; Fig. 2c) compared to deeper layers (*P*-value < 0.05, Mann-Whitney U test), where their relative abundance remained relatively constant, except at some discrete depths (e.g., 149–152 cmbsf, 275–278 cmbsf, 539–542 cmbsf, and 644–647 cmbsf). Correlation analysis showed a significant positive correlation between the summed relative abundance of all alkane-degrading genes and total *n*-alkane concentration (Fig. S3a; *P*-value < 0.01, Spearman rank correlation), suggesting that substrate availability influences the distribution of alkane degraders in the hadal subsurface sediments. The vertical variation of *alkB* was consistent between metagenomic analysis and qPCR quantification, as reflected by a high correlation (*P*-value <0.01, Spearman rank correlation; Fig. S3b). Notably, genes involved in short-chain alkane oxidation (*prmAC*, *pbmoABC,* and *pbmoXYZ* for propane and butane degradation) were largely absent (Fig. 2A) (23).

**Fig 3:**
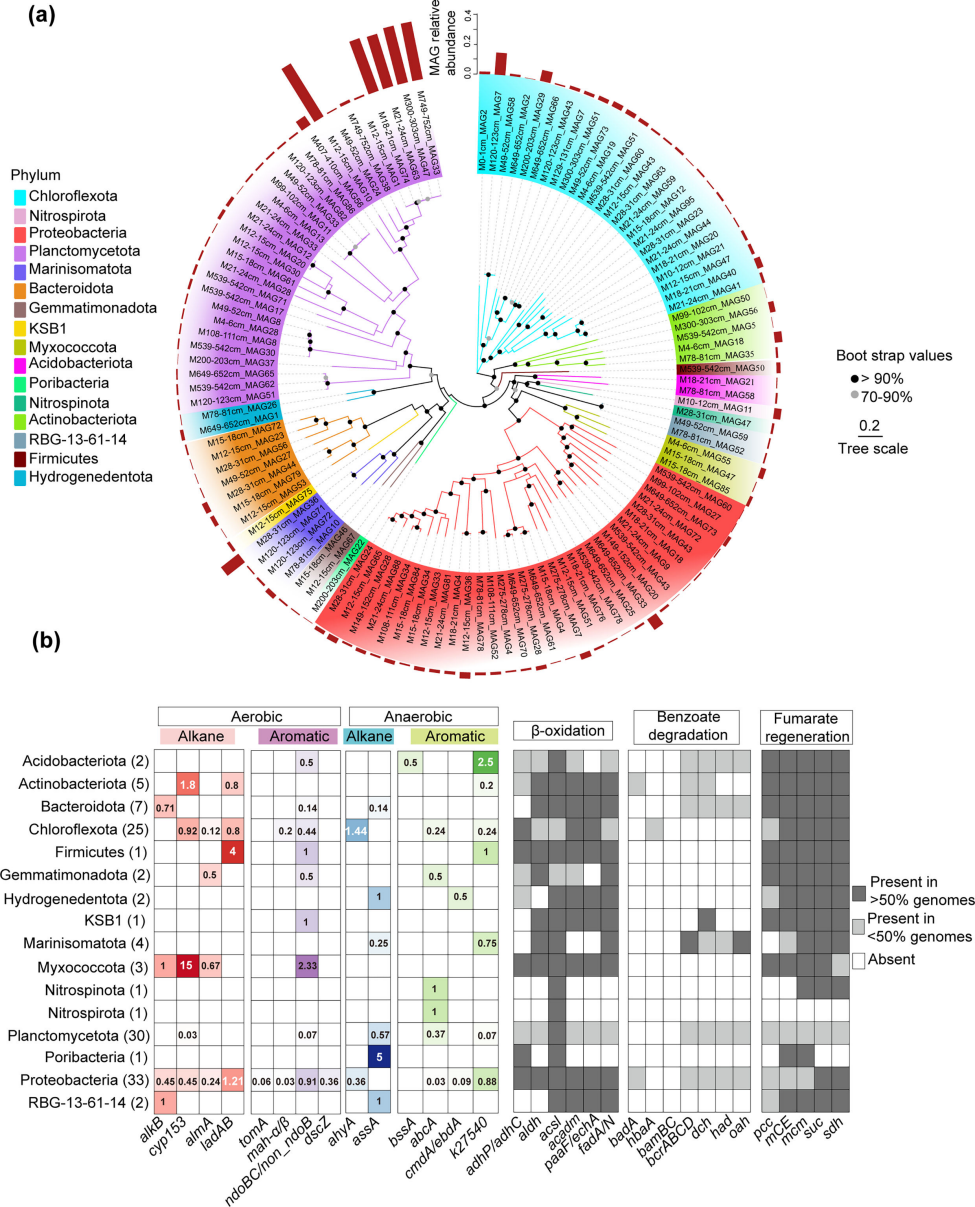
Predicted metabolic models for degradation of hydrocarbons based on bacterial MAGs harboring diverse hydrocarbon degrading-related genes. (**a**) Maximum-likelihood phylogenetic tree of 120 MAGs containing putative hydrocarbon degradation genes and their relative abundance in the sediment core. (**b**) Occurrence of protein-encoding genes putatively involved in pathways for degradation of several kinds of hydrocarbons in MAGs. Values in brackets indicate the number of MAGs within each taxonomic unit. The numbers in each small cell represent the corresponding gene counts.

Genes potentially encoding enzymes involved in anaerobic hydrocarbon degradation were significantly more abundant than those involved in aerobic degradation (*P*-value < 0.05, paired *t*-test based on CLR-transformed data; Table S5; Fig. 2c). Among these, putative *abcA*-like homologs (involved in benzene degradation) and putative *k27540*-like homologs (involved in naphthalene degradation) were the dominant aromatic hydrocarbon-degrading enzyme-encoding genes (Fig. 2a). Additionally, genes potentially associated with aerobic naphthalene degradation (*non_ndoB* and *nodB*) and the monoaromatic dioxygenase gene *mah-*αβ (targeting benzene/toluene) were also detected. Anaerobic hydrocarbon-degrading microorganisms in sediments can potentially catalyze alkane degradation via two pathways, the first via AhyA (24), and putative genes encoding this enzyme were present in some Chloroflexota and Proteobacteria species (Fig. 3b). The second is via addition of fumaric acid catalyzed by AssA (Fig. 3b) (25). The genes potentially involved in anaerobic alkane degradation, *assA* and *ahyA*, exhibited variation trends opposite to those involved in aerobic degradation. Their abundances were lowest at 21 cm below seafloor (cmbsf), followed by a gradual increase with depth (Fig. 2d). These findings suggest a transition from aerobic alkane degradation in upper sediment layers to anaerobic processes in deeper sediments, where oxygen is typically depleted (26).

### Dominance of hydrocarbon degraders in the subsurface microsphere

To assess the taxonomic distribution of HYD genes, we compared the 2,400 gene sequences against the NCBI-nr database (Fig. S4, Tables S4 and S5). The putative HYD genes affiliated with Chloroflexota accounted for the highest proportion (11.18%) (Fig. S4, Table S5), with the majority in the classes Dehalococcoidia, Anaerolineales, Thermoflexales, and the SAR202 cluster. Other significant bacterial groups containing putative HYD genes included Proteobacteria, Planctomycetota, Actinomycetota, and Bacteroidota. A smaller fraction (0.58%) of archaea, including Bathyarchaeota, Lokiarchaeota, and Thorarchaeota*,* contained genes likely associated with anaerobic hydrocarbon degradation (Fig. S4, Table S5). Taxonomic profiling revealed these phyla as dominant groups in the sediments, as confirmed by both metagenomic (Fig. S5b) and 16S rRNA gene sequencing data (2). This correlation suggests that the microbial groups most abundant in the sediment core were also the primary hydrocarbon degraders.

A total of 342 dereplicated MAGs, each with >75% completeness and <10% contamination, were recovered from the combined metagenomic data set (Table S6). Of these, 120 MAGs (~35.1%) from 16 bacterial phyla (Chloroflexota, Proteobacteria, Planctomycetota, Marinisomatota, Bacteroidota, Actinobacteriota, Myxococcota, Acidobacteriota, Hydrogenedentota, RBG-13-61-14, Firmicutes, KSB1, Nitrospinota*,* Nitrospirota, and Poribacteria) harbored genes potentially encoding enzymes in hydrocarbon-degrading processes, with an average of 3.52 copies per genome (Fig. 3; Table S7). Their functional annotation was supported by phylogenetic clustering of HYD genes in MAGs with experimentally confirmed genes (Fig. S6a through f). Notably, the phyla Poribacteria, Nitrospinota, Nitrospirota, RBG-13-61-14, Gemmatimonadota, and Hydrogenedentota have not previously been implicated in hydrocarbon degradation.

MAG-encoded HYD genes exhibited distinct substrate preferences among different microbial groups (Fig. 3b). Except for Nitrospinota and Nitrospirota (encoding only *abcA*-like homologs, potentially involved in benzene oxidation), Poribacteria (encoding only *assA*, potentially involved in anaerobic alkane oxidation), and KSB1 (encoding only *ndoBC*, potentially involved in naphthalene oxidation), all other groups encoded diverse HYD genes. For instance, Acidobacteriota contained genes for enzymes putatively involved in aromatic hydrocarbon degradation (*k27540*-like homologs and *non_ndoB*), while RBG-13-61-14 contained genes for those putatively involved in alkane degradation (*alkB* and *assA*). Hydrogenedentota and Marinisomatota each encoded two distinct gene types encoding enzymes likely involved in anaerobic hydrocarbon degradation: *assA* and *cmdA* and *assA* and *k27540*-like homologs, respectively. The remaining groups contain a broader range of possible hydrocarbon oxidation genes. For example, species in the phyla Actinobacteria, Firmicutes, Myxococcota, and Bacteroidota contained genes likely encoding enzymes for degrading both aromatic compounds and alkanes. However, the higher number of putative alkane-degrading gene copies in these phyla suggests a preference for alkane degradation (Fig. 3a; Table S7). Chloroflexota, Proteobacteria, and Planctomycetota encoded more diverse genotypes, allowing for a wider substrate range. Notably, Chloroflexota preferentially degrade *n*-alkanes under anaerobic conditions and encode multiple putative *ahyA* genes (averaging 1.44 copies per genome), whereas aerobic alkane degradation is primarily carried out by Proteobacteria, which in this sample encode multiple putative *ladAB* genes (averaging 1.22 copies per genome).

Importantly, these HYD groups not only contained genes likely involved in the initiation of hydrocarbon degradation but also associated downstream metabolic genes. For instance, nearly all aerobic hydrocarbon degradation groups contained putative genes encoding a complete β-oxidation pathway (Fig. 3b). KSB1 contains putative *ndoBC* genes and all putative essential genes required for fumaric acid synthesis (Fig. 3b). Previous studies have demonstrated that KSB1 participates in aromatic degradation via fumaric acid addition (27), further supporting our findings. These results suggest that HYD microorganisms in sediments exhibit broad substrate selection and utilization capacity, mediated by a diverse array of HYD genes. Furthermore, the MAGs containing the five predominated HYD gene categories (*cyp153*, *nodB/non_nodB*, *ahyA*, *assA*, and *abcA*-like homologs) (Fig. S7) displayed distribution patterns similar to those of the respective gene categories across the sediment core (Fig. 2c).

Spearman’s correlation analysis was performed between the top 50 dominant orders and environmental factors (Fig. S8). Among these, 28 orders exhibited a positive correlation with total *n*-alkane concentrations, implicating widespread involvement of sediment-associated microorganisms in alkane metabolism (*P*-value < 0.01). These included Rhodobacterales, Steroidobacterales, Oceanospirillales, and Pseudomonadales from the phylum Proteobacteria; CCM11a and Phycisphaerales from the phylum Planctomycetota; SAR202 and Anaerolineales from the phylum Chloroflexota, as well as Omnitrophales, Hydrogenedentiales, Cytophagales, Dadabacteriales, norank_c__BD2-11_terrestrial_group, Nitrosopumilales, and Woesearchaeales. Interestingly, the abundance of these microbial groups correlated positively with NO_2_^−^ and NO_3_^−^ concentrations and negatively with NH_4_^+^ concentrations in the sediments. This finding suggests that NO_3_^−^ likely serves as an electron acceptor for anaerobic hydrocarbon degraders in the deep subseafloor. Supporting this, the *narG* gene, responsible for dissimilatory NO_3_^−^ reduction, was identified in diverse taxa including Planctomycetota, Marinisomatota, and Chloroflexota (Table S8).

### Metabolic characteristics of dominant HYD microorganisms in hadal sediments

**Fig 4:**
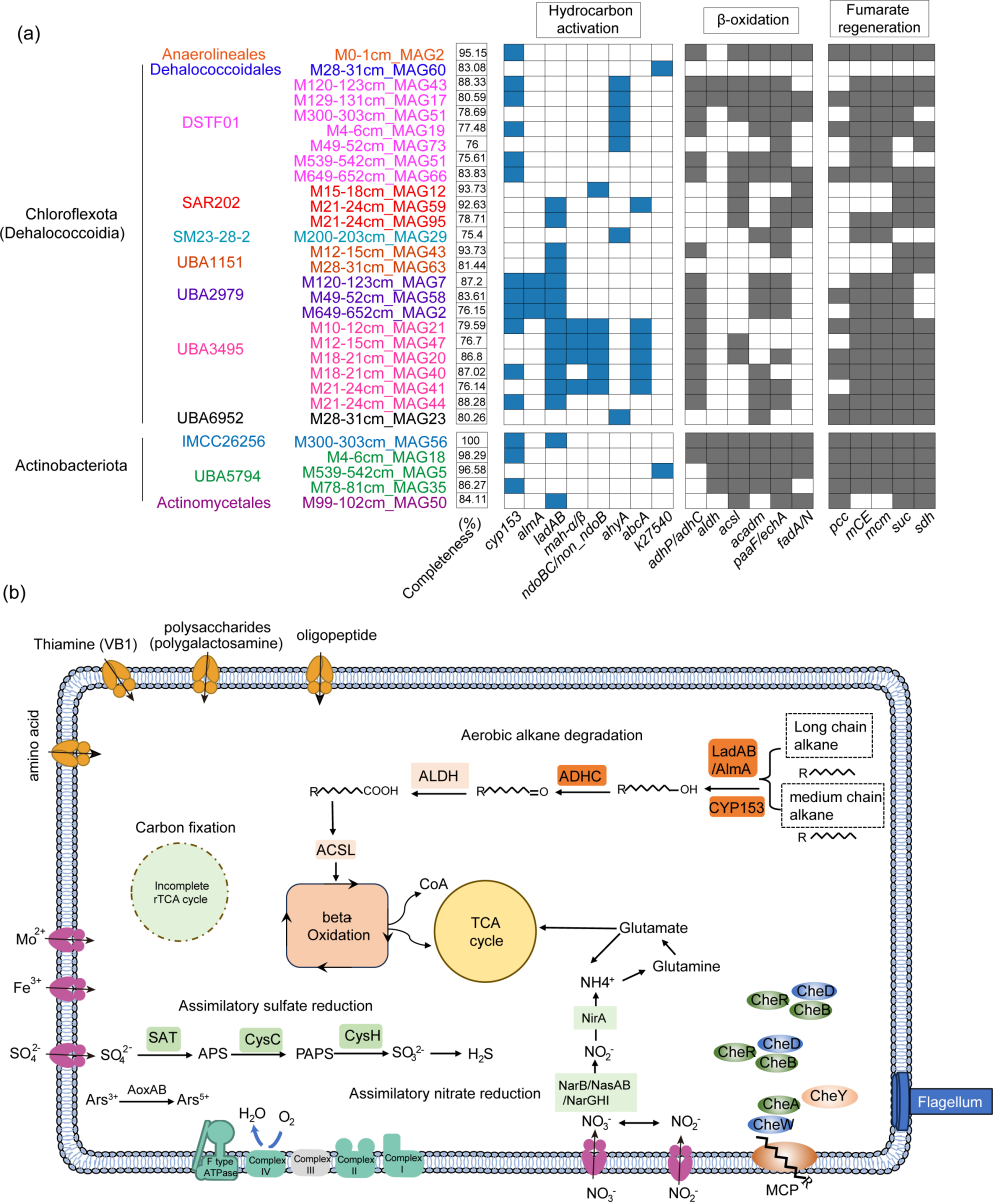
Hydrocarbon degradation characteristics of the phyla Chloroflexota and Actinobacteriota in the sediment core MT20-750. (**a**) Putative HYD genes in the MAGs from phyla Chloroflexota and Actinobacteriota. (**b**) Metabolic pathway reconstruction of the order UBA2979 in the phylum Chloroflexota.

The potential hydrocarbon degradation capacity of Chloroflexota, the most abundant microbial phylum in sediments, was further analyzed (Fig. 4). The HYD MAGs within Chloroflexota comprise nine orders, each harboring distinct putative HYD genes likely encoding enzymes involved in aerobic alkane degradation (Fig. 4a). For instance, o_DSTF01 contained *cyp153* and *ahyA*, o_UBA2979 contained *cyp153*, *almA,* and *ladAB*, while Anaerolineales contained *cyp153*. Notably, the order UBA2979 has not been shown to possess hydrocarbon oxidation capabilities. To further investigate its hydrocarbon-degrading capacity, the metabolic pathways of three UBA2979 MAGs (M120-123cm_MAG7, M49-52cm_MAG58, and M649-652cm_MAG2) were analyzed (Fig. 4b). Their genomic completeness ranged from 76.2% to 86.2%, with an average relative abundance of 5.7%. These organisms contained multiple copies of putative alkane degradation genes (*cyp153*, *ladAB,* and *almA*) alongside a complete β-oxidation pathway. They also contained genes encoding putative methyl-accepting chemotaxis proteins (MCPs) with a potential role in detecting environmental alkanes and genes encoding some putative enzymes in an incomplete reverse TCA (rTCA) pathway, suggesting the potential for carbon fixation. To validate the alkane oxidation capability of order UBA2979, we cloned one *almA* gene (MDBPCMKO_01555) found in M49-52cm_MAG58 from this order into an expression vector (pRSET-A) downstream of the ferredoxin and reductase genes (MDBPCMKO_00267 and MDBPCMKO_00787) and transformed this plasmid into *Escherichia coli* BL21(DE3) pLyS host cells to verify its functionality (Fig. S9a and b). The recombinant strain could degrade 6%–13% C_18–36_ alkanes within 36 h and 38% of C_18_ and C_19_ alkanes within 72 h (Fig. S9c and d), demonstrating this gene encodes a functional alkane degradation enzyme.

Five of the seven o_DSTF01 MAGs contained putative *ahyA* genes encoding enzymes catalyzing anaerobic alkane hydroxylation at the C2 position (Fig. 4a). Subsequent oxidation of the hydroxyl group yields a ketone (28), while carboxylation at C3 produces 2-acetylalkanoic acid (29, 30). We systematically examined two *ahyA* genes (*ahyA120_123_580* and *ahyA120_123_587*) from o_DSTF01, alongside two *ahyA* reference genes in *Desulfococcus oleovorans* (accession no.: Oqx63933) and *D. oleovorans* Hxd3 (accession no.: WP_012173623) (25, 26, 31) (Fig. S10 and S11). The 3D structure of these proteins predicted by Alphafold3 was docked into the *n*-C_10_ and *n*-C_20_ 3D conformers. Each protein features an elongated hydrophobic tunnel lined with aromatic residues, suitable for accommodating aliphatic chains. All docking complexes yielded negative binding energies, with consistently lower energies (or higher affinity) for *n*-C_10_ than for *n*-C_20_ (Table S9). Among the four proteins, AhyA120_123_587 displayed the strongest binding affinity. Its binding mode aligned with the canonical mechanism of anaerobic alkane hydroxylases, and its enhanced affinity is likely due to a more favorable pocket geometry and a dual-arginine motif that positions the substrate at the catalytic site. Analysis of the catalytic site identified key residues (GLU-583, ARG-45, ARG-585, and SER-587) that form hydrogen bonds with *n*-C_10_ and likely constitute the catalytic core for hydroxylation.

The dominant phylum Proteobacteria possessed the genetic potential to degrade multiple types of hydrocarbons (Fig. S4 and S12). Diverse putative HYD genes such as *ladAB*, *ndoBC*, *abcA*-like homologs, and *k27540*-like homologs were detected in different orders of Alphaproteobacteria, including Rhizobiales, Rhodospirillales, Sphingomonadales, SMXQ01, as well as some unclassified lineages. Specifically, MAGs of SMXQ01 and Sphingomonadales contained at least one putative benzene- or naphthalene-degrading gene (*abcA*-like homologs, *ndoBC*, or *k27540*-like homologs). Rhizobiales contained a putative *almA*, indicating a potential capacity for LC-alkane degradation. Furthermore, members of Rhodospirillales (e.g., M28-31cm_MAG43) exhibited a notable enrichment of *k27540-*like homologs (up to 17 copies) associated with naphthalene degradation (Table S7), consistent with a previous report that this order can degrade polycyclic aromatic hydrocarbons (PAHs) (32). Putative HYD genes displayed greater diversity in Gammaproteobacteria (including orders such as Burkholderiales, Enterobacterales, Pseudomonadales, Woeseiales, Xanthomonadales, and clades GCA-001735895 and MnTg04) than in Alphaproteobacteria (Fig. S12). Genes encoding enzymes likely involved in aerobic hydrocarbon degradation were broadly distributed among these MAGs, whereas those likely associated with anaerobic pathways were comparatively rare. This supports the view that Gammaproteobacteria preferentially utilize aerobic processes for hydrocarbon degradation.

Actinobacteriota*,* the second abundant putative HYD phylum in the hadal sediment, were primarily represented by the orders IMCC26256, UBA5794, and Actinomycetales. They are putative mid- and long-chain *n*-alkane degraders, utilizing enzymes encoded by *cyp153* and *ladAB* (Fig. 4a). For example, M300-303cm_MAG56 (100% genome completeness) encoded seven putative *cyp153*, one *ladB*, and all the key genes encoding the β-oxidation pathway (Fig. 4a). Thus, Actinobacteriota likely play a role in driving hydrocarbon metabolism in deep-sea trench sediments. The genus *Brevibacillus* (phylum Firmicutes) reportedly degrades a range of hydrocarbons, including pyrene, benzo(a)pyrene, naphthalene, anthracene, and *n*-alkanes (33–39). From this phylum, we identified *Brevibacillus agri* M539-542cm_MAG50, which contained four putative LC-alkane degrading genes (*ladAB*, C_15–36_ alkane oxidation), one *k27540-*like homolog (potentially involved in naphthalene oxidation), one *ndoB* gene (potentially involved in naphthalene oxidation), and all the key genes encoding enzymes involved in β-oxidation and fumarate regeneration (Fig. 4a; Table S7). The average relative abundance of this MAG across the sediment core was 2.6%. Its metabolic model was further predicted and reconstructed based on the metabolic processes of other long-chain alkane-degrading bacteria (40, 41) (Fig. S13). Three MAGs from the phylum Myxococcota (M15-18cm_MAG47, M15-18cm_MAG85, and M4-6cm_MAG55) encoded putative *cyp153* (14 to 16 gene copies), *almA* (1, 0, and 1 copy, respectively), and naphthalene dioxygenase *ndoB/non_ndoB* (6, 0, and 0 copies, respectively) genes, with an average relative abundance of 2.17%. Most *Bacteroidota* MAGs contained putative *alkB* genes.

Poribacteria have only been found with high microbial diversity and abundance in shallow water sponge species (42) and have no known HYD capacity. Herein, we obtained a Poribacteria MAG (M200-203cm_MAG22) with 91.86% completeness and 5.68% contamination, showing a relative abundance of ~2.6%. This MAG contained five putative *assA* genes, which encode enzymes involved in anaerobic alkane degradation, along with genes involved in fumarate regeneration and β-oxidation (Table S7). Genome analysis revealed that this bacterium did not encode any oxygen-utilizing proteins, such as cytochrome oxidase; therefore, it is likely anaerobic. In addition, it can potentially fix CO_2_ via the Wood-Ljungdahl (WL) pathway (Fig. S14). This suggests this Poribacteria bacterium may be a facultative heterotrophic anaerobic HYD microorganism.

Other microorganisms with the potential capacity of degrading hydrocarbons were identified in the core. Two MAGs from the phylum Acidobacteriota contained *non_nodB* and *K27540-*like homologs and other putative benzoate degradation-related genes (*bcrABCD*, *dch*, *had,* and *oah*), indicating their potential to degrade naphthalene (Fig. 3b). The KSB1 genomes from hydrothermal sediments contained genes involved in anaerobic degradation of hydrocarbon and activating polycyclic aromatic hydrocarbons (PAHs) and alkanes via fumarate addition (27, 43). The KBS1 MAGs obtained in the core showed similar characteristics, encoding the putative naphthalene-degrading gene *non_ndoB*.

To investigate the broader biogeochemical roles of these HYD microorganisms, we profiled genes related to the carbon, nitrogen, and sulfur cycles in 120 HYD MAGs (Fig. S15). The genetic repertoire suggested that many of these taxa were genetically equipped to perform critical redox processes beyond hydrocarbon activation, including denitrification and dissimilatory sulfate reduction. The presence of these pathways indicated their capacity to utilize electron acceptors such as nitrate, nitrite, and sulfate, which is essential for sustaining anaerobic respiration and, consequently, hydrocarbon turnover in the energy-limited hadal biosphere. Furthermore, the genomic potential for carbon fixation via the WL pathway in Nitrospirota, Planctomycetota, and RBG-13-61-14 suggested that, in addition to utilizing *in situ* organic matter, these taxa can also support autotrophic growth.

### Bacterial isolates capable of hydrocarbon degradation

Having established the metabolic potential of hadal HYD bacteria through metagenomic analyses, we next examined the phenotypic traits of cultivable isolates and their phenotype-genotype congruence. A total of 131 potential HYD strains from 23 sediment depths were isolated using media with mixed alkanes (C_6_, C_11_, C_16_, C_19_, C_22_, C_25_, C_28,_ and C_32_) as the sole carbon source at 16°C (Fig. S16a and b). These strains were mainly affiliated with Actinobacteriota (77 strains, 58.7%), followed by Proteobacteria (43 strains, 32.8%) and Firmicutes (11 strains, 8.4%) (Fig. S16c). We also isolated 69 potential petroleum-degrading strains on media containing petroleum crude oil:diesel oil (1:4 [M/M]) as the sole carbon source at 16°C. These bacteria belonged to the same three phyla mentioned above (Fig. S16c). All the isolates belonged to 20 classes and 50 genera, among which 38 genera have been reported to possess HYD ability (Table S10). Actinobacteriota were the most abundant and contained potential mid- and long-chain *n*-alkane degradation genes (*cyp153* and *ladAB*) (Fig. 4a). Among these, one MAG (M99-102cm_MAG50), identified as *Microbacterium indicum*, harbored two putative *ladA* long-chain *n*-alkane-degrading genes (Fig. 4a), suggesting its potential for long-chain alkane degradation. Supporting this, three isolates of *Microbacterium* (*M. esteraromaticum* HXX-185, *M. keratanolyticum* HXX-163, and *M. yannicii* HXX-198) exhibited greater ability to degrade mid-chain and long-chain *n*-alkanes of C_16_, C_19_, and C_32_ over the short-chain C_6_ and C_7_ compounds (Fig. S16d), supporting a correlation with their predicted genotypic profile.

**Fig 5:**
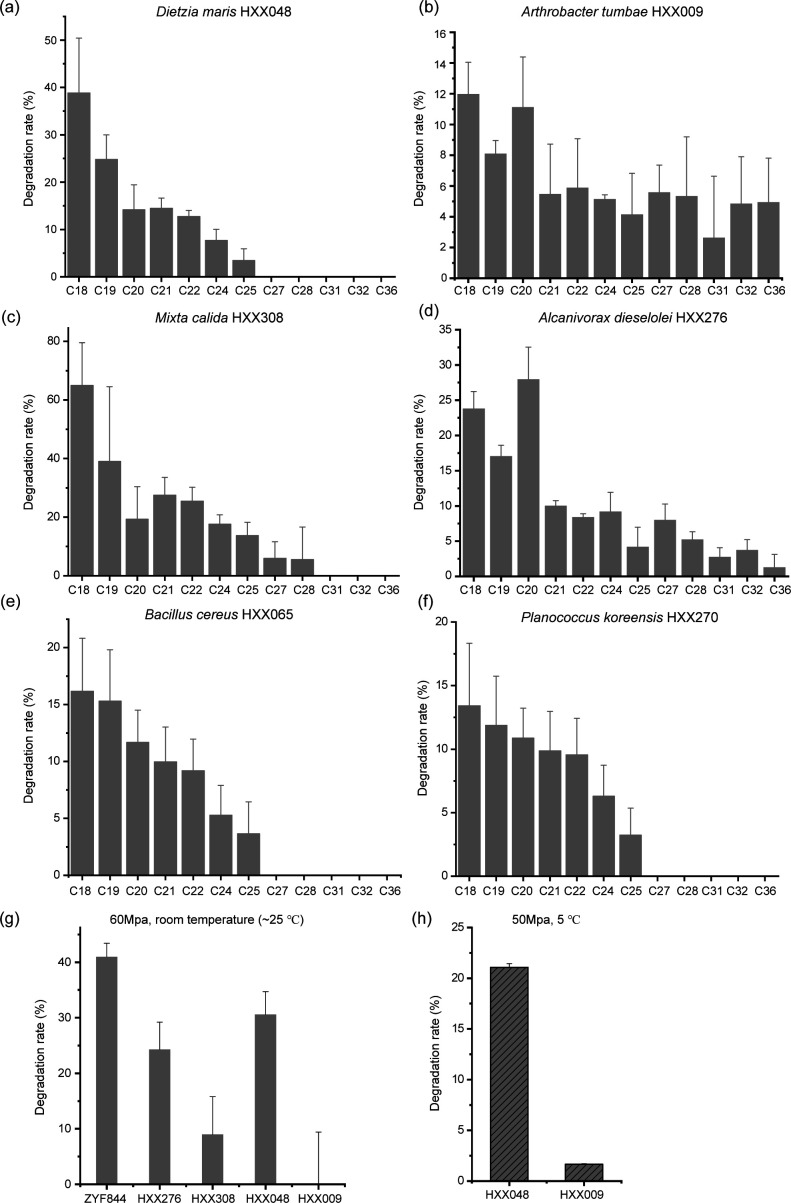
The hydrocarbon-degrading ability of representative bacterial isolates under low temperature and high pressure. The *n*-alkane-degrading ability of two Actinomycetota isolates (**a and b**), two Pseudomonadota isolates (**c and d**), and two Firmicutes isolates (**e and f**) cultured at 5°C and 0.1 MPa (atmospheric pressure) for 30 days. The *n*-eicosane-degrading ability of representative bacterial isolates cultured at room temperature (~ 25°C) and high pressure (50 MPa) for 30 days (**g**). The *n*-eicosane-degrading ability of representative bacterial isolates cultured at 5°C and high pressure (50 MPa) for 45 days (**h**).

Two isolated strains from each of three phyla were selected to verify their *n*-alkane degradation ability over 30 days (Fig. 5). At 5°C and 0.1 MPa, all strains could degrade mid-chain and long-chain (C_18–36_) *n*-alkanes, with the efficiency typically increasing with reduced carbon chain length (Fig. 5a through f). At 25°C and 60 MPa, four strains, *A. jadensis* ZYF844 (a positive reference strain with confirmed alkane-degrading ability) (4), *Dietzia maris* HXX048, *Alcanivorax dieselolei* HXX276, and *M. calida* HXX308, degraded *n*-eicosane (C_20_) (Fig. 5g). At a physiologically relevant temperature (5°C) and pressure (50 MPa), *D. maris* HXX048 and, to a lesser extent, *Arthrobacter tumbae* HXX009 could utilize *n*-eicosane as its sole carbon source (Fig. 5h). Collectively, this shows some species can degrade hydrocarbons under the extreme conditions of the deepest hadal sediments.

### Identification of potential hydrocarbon-synthesizing bacteria

Considering previous evidence that unknown heterotrophic organisms may contribute to hydrocarbon production in hadal surface sediments (4), we hypothesized that hydrocarbon production and consumption occur simultaneously, forming a potential hydrocarbon cycle in hadal sediments. To test this, the genetic potential for hydrocarbon synthesis was examined in hadal benthic microorganisms. We detected putative *oleBC* and *oleC* genes, potentially encoding olefin biosynthetic enzymes, in 46 MAGs (Table S11), spanning multiple phyla and classes, including Planctomycetota, Verrucomicrobiota, Deltaproteobacteria (Desulfobacterota, RBG-13-61-14, and Myxococcales), Acidobacteriota, Actinobacteriota, Elusimicrobiota, Gemmatimonadota, Hydrogenedentota, Nitrospinota, and Gammaproteobacteria. Among these, 18 MAGs were identified as potential hydrocarbon-degrading groups (Table S11, marked in red). The relative abundance of MAGs harboring potential hydrocarbon synthesis genes showed a pattern similar to the variation in alkane concentrations, although no significant correlation was found. In contrast, a significant correlation (*P* < 0.05) was observed between hydrocarbon synthesis and alkane degradation genes (Fig. 2b), suggesting that alkanes consumed in the hadal sediments may be derived from *in situ* synthesis by specific microorganisms.

## DISCUSSION

### Abundant hydrocarbons and HYD microorganisms in trench sediments

Hydrocarbons, including alkanes and aromatics, are produced and distributed across various marine sedimentary environments, such as coastal zones, hydrothermal vents, cold seeps, polar regions, and hadal trenches (4, 44–48). Microbial biodegradation drives hydrocarbon metabolism in sediments (19, 41). Here, we reported the first large-scale depth profile (0–752 cmbsf) of *n*-alkane concentrations and major HYD microbial groups in hadal sediments from the deepest site of the ocean (10,816 m).

Total concentrations of *n*-alkanes (C_18–36_) in this core varied from 310 to 8,724 ng/g (average = 2,570.17 ng/g), which is comparable to or slightly higher than that in coastal and other deep-sea sediments (Table S12) (49–51). For instance, the *n*-alkane (C_15–35_) concentration in the coastal marine sediments of China was 120–1,680 ng/g (average = 560 ng/g) (50). However, the concentrations of *n*-alkane in petroleum seepage deep-sea sediments from the Eastern Gulf of Mexico were 1,045–2,845.3 ng/g (46), which is comparable to the less polluted sediment core investigated in this study (2,750 ng/g). Anaerobic hydrocarbon degradation processes were found in polar and subpolar coastal environments where the concentration of aliphatic hydrocarbons ranged from 27 to 5,000 ng/g (47). High concentrations of *n*-alkanes were detected in other trench environments. For example, 341–1,685 ng/g of *n*-alkanes (C_16–22_) was detected in surface sediments of the southern Mariana Trench (4,900–7,068 m depth) (18), while sediments deeper than 10,000 m showed average *n*-alkane concentrations of ~2.3 µg/g (4). In the hadal sediment samples from the Kermadec and Atacama Trenches (52) and the Yap Trench at depths of 5,058 m and 6,578 m, the highest alkane concentrations in the surface layer were 71.74 μg/g (53). The concentration of *n*-alkanes in the slope sediments is likely higher than at the very bottom of the trench. Specifically, the sediment core MT20-750 studied in this study showed a clear shift in sedimentation patterns in the upper 28 cmbsf compared to the layers below, which is presumed to be due to a sudden deposition of slope sediment (4) (Fig. 1a).

The high concentration of *n*-alkanes was accompanied by abundant HYD genes detected across the 22 metagenomic samples (Fig. 2b), suggesting that hydrocarbons serve as available carbon sources for hadal benthic microbial communities. Previous studies have highlighted aromatic compound utilization as a key adaptation of hadal microorganisms (7). Consistently, diverse genes encoding enzymes potentially involved in aromatic degradation were detected in the sediment samples. However, their abundances were lower than those of putative alkane-degrading genes, suggesting *n*-alkanes likely play a more important role in sustaining hadal benthic HYD communities. The absence of detectable aromatic compounds in the sediments further supports this hypothesis (Table S1). Putative anaerobic alkane-degrading genes were more abundant, especially in sediment layers below 30 cmbsf, likely attributable to redox transitions.

We demonstrated the broad distribution of HYD microorganisms across trench environments by expanding the analysis using publicly available metagenomes from the Mariana Trench (14) and Atacama Trench (54). Comparable to observations in this study, high abundance and diversity of HYD genes were detected in these samples (Fig. S17 and S18), with significantly higher HYD gene abundance in hadal than in abyssopelagic sediments (Fig. S17). Together with the diverse set of genes involved in alkane and aromatic compound degradation (including *alkB*, *baiCD*, *benA*, *bsh*, *cutC*, *etnE*, and *P450*) detected in the Yap Trench sediments (48), these results suggest that microbially driven hydrocarbon degradation is ubiquitous across different hadal trench sediments. This was further confirmed by mapping our MAGs to publicly available Challenger Deep metatranscriptomic data (14), which revealed active expression of the abundant HYD genes, including *cyp153-*, *almA-*, *non_ndoB-*, *ahyA-*, and *abcA*-like homologs (Fig. S19).

Taxonomical analysis of the dominant HYD microorganisms revealed that the sediment core was primarily inhabited by members of the phyla Chloroflexota, Pseudomonadota, Actinomycetota, Planctomycetota, Acidobacteriota, Bacteroidota, and Firmicutes (Fig. 3). This community composition differed markedly from that previously observed in the overlying seawater (4). In these waters (~10,000 m depth), the order Oceanospirillales was significantly enriched compared to shallower layers, with key HYD genera including *Oleibacter*, *Thalassolituus*, and *Alcanivorax* (4). In contrast, both 16S rRNA gene sequencing (2) and enrichment cultures from this study detected these Oceanospirillales genera at very low abundance in hadal sediments, suggesting either limited availability of their preferred hydrocarbon substrates or an inability to thrive under sedimentary conditions.

The extreme environmental conditions of the hadal zone, including high pressure, low temperature, and darkness, have selected for novel microbial lineages with unique adaptive strategies (7, 14). In line with this, we identified several previously unrecognized HYD groups in the sediment core (Table S7). While Chloroflexota are known for degrading aromatic compounds (e.g., benzoate and polyaromatic hydrocarbons) (12), we found that some species also possessed the *alma* gene, suggesting an additional capacity to degrade long-chain C₁₈–₃₆ *n*-alkanes (Fig. S9). Among Proteobacteria, the dominant putative hydrocarbon degraders were affiliated with Gammaproteobacteria and Alphaproteobacteria. The former likely serves as key aerobic degraders of both alkanes and aromatics, while the latter, particularly the order Rhodospirillales, likely performs anaerobic aromatic degradation, as evident by their multiple relevant gene copies (Table S7) and consistent with prior reports of PAH-degrading capability (55). This functional potential was confirmed by cultivation experiments, which yielded various proteobacterial strains with different substrate preferences (56). Deltaproteobacteria are typically prominent anaerobic hydrocarbon degraders (57). However, they were scarce in the hadal sediments. This is likely because the prevalent nitrate and nitrite, being thermodynamically more favorable than sulfate, suppressed sulfate reduction (58), thereby limiting the niche for these sulfate-reducers. These findings suggest the partitioning of hydrocarbon niches among different Proteobacteria classes in the hadal sediment.

Our results further expanded the taxonomic diversity of hydrocarbon degraders by identifying Poribacteria, Nitrospinota, Nitrospirota, RBG-13-61-14, Gemmatimonadota, and Hydrogenedentota as potential phyla capable of this process. The possible presence of a full WL pathway for carbon fixation in Poribacteria suggests metabolic versatility and a “feast-or-famine” strategy, enabling it to degrade alkanes when organic carbon is abundant or switch to autotrophy under carbon-limited conditions. This mixotrophy is likely a common adaptive strategy for energy and carbon acquisition in the hadal biosphere (7, 22). Additionally, these bacterial groups exhibited the capacity to use a range of electron acceptors, e.g., nitrate or sulfate (Fig. S8), suggesting the presence of diverse HYD pathways. These findings aligned with previous reports that sulfate-reducing and denitrifying bacteria can anaerobically degrade alkanes and aromatic hydrocarbons using sulfate and nitrate as electron acceptors (59–61). Because oxygen levels drop rapidly from oxic conditions in the upper 0–30 cmbsf to microxic or anoxic conditions below (62), facultative anaerobes may switch between aerobic and anaerobic respiration depending on oxygen availability. These observations suggest that multiple oxygen-dependent and oxygen-independent pathways contribute to hydrocarbon transformation in hadal sediments. However, the precise mechanisms linking hydrocarbon oxidation with nitrate or sulfate reduction remain to be clarified.

Aromatic hydrocarbons are another group of persistent organic pollutants in nature that can accumulate in deep-sea sediments (63, 64). The concentrations of 16 EPA-priority PAHs varied from 2.0 to 41.6 ng/g in the deep-sea sediments of the high-latitude Arctic Ocean (65), and phenanthrene was most abundant *in situ*. Predicted PAH degraders, including *Cycloclasticus*, *Pseudomonas*, *Pseudoalteromonas*, *Halomonas*, *Marinomonas,* and *Dietzia*, had the most important role in PAH mineralization *in situ* (65). However, the concentration of aromatic hydrocarbons in hadal sediments has not been previously measured. In this study, numerous aromatic hydrocarbon-degrading genes were detected in hadal deep sediment, including *nodB*, *non_ndoB*, *MAH-*αβ*,* and *dscZ* (encoding enzymes involved in aerobic degradation), as well as *abcA*-like homologs and *k27540*-like homologs (encoding enzymes involved in anaerobic degradation). While Xiao et al. (7) highlighted aromatic compound utilization as a key adaptation of hadal microorganisms (7), our study revealed that genes likely involved in aromatic hydrocarbon degradation are less abundant than those likely associated with alkane degradation. This suggests that the role of alkane degradation in hadal sediments may have been previously underestimated. The ecological role of aromatic compound metabolism in hadal sediments may be more variable than previously thought and warrants further investigation.

### Hydrocarbon metabolism in the Challenger Deep

**Fig 6:**
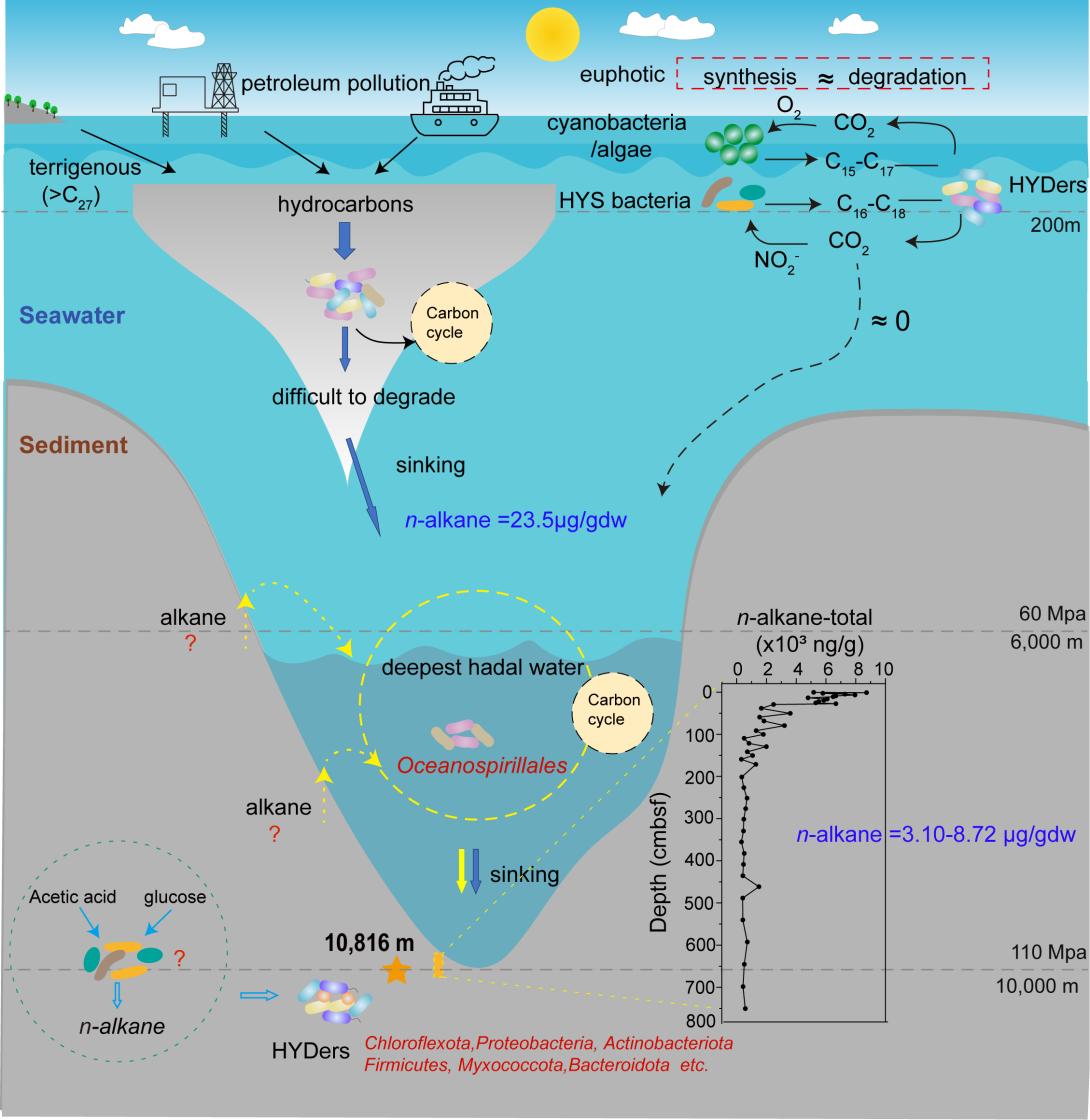
Hydrocarbon metabolism in the hadal environment of the Challenger Deep. Hydrocarbons in the ocean are derived from natural seepage, anthropogenic inputs, terrigenous sources, and biological production by cyanobacteria and eukaryotic phytoplankton. In the euphotic zone, cyanobacteria and phytoplankton produce short- to medium-chain *n*-alkanes (C_15–24_), which are rapidly degraded by co-occurring microbes. In contrast, terrestrially derived long-chain *n*-alkanes (C_25–35_) and hydrocarbons from natural seeps or human activities undergo extensive but incomplete consumption in the upper water column, leaving recalcitrant fractions to sink into the deep and reach the hadal depths. Hydrocarbon degradation in the hadal water is primarily mediated by obligate hydrocarbonoclastic bacteria, notably certain members of the Oceanospirillales (e.g., *Oleibacter*, *Thalassolituus*, *Alcanivorax*, and *Oleiphilus*), whereas hadal sediments are dominated by facultative hydrocarbon degraders, including Chloroflexota, Proteobacteria, and Actinobacteriota. Additional hydrocarbon inputs to hadal sediments may also originate from *in situ* biosynthesis by heterotrophic bacteria and subsurface petroleum seepage.

Hydrocarbons in the ocean may originate from natural oil seeps, human activities, and terrigenous input and are also produced by cyanobacteria*,* eukaryotic phytoplankton, and higher terrestrial plants (66, 67). Natural oil seeps and human activities release 0.47–8.3 million tons of petroleum annually (17). While cyanobacteria and eukaryotic phytoplankton generate around 100–500 times greater quantities of hydrocarbons in the ocean compared to natural seeps and anthropogenic sources (15, 17), they are mostly short- to mid-chain *n*-alkanes (C_15–24_), with C_15_, C_17,_ and C_19_ predominating (16, 68). However, due to rapid biodegradation by colocalized hydrocarbon-degrading microbiomes, only a small fraction of these compounds accumulate in the deeper water column (Fig. 6) (15), predominantly terrestrial plant-derived long-chain *n*-alkanes C_25–35_ with a strong odd-to-even carbon preference (69). Unlike the *n*-alkanes found in the sinking particles in the overlying water column (2,000–6,000 m) and the upper sediment layers at 10,000 m depth in the Challenger deep (4), short-chain alkanes (C_14–17_) are absent in the MT20-750 sediment core, with medium-chain alkanes (C_18–24_) being predominant and exhibiting a clear even-carbon preference (Table S13). The carbon and hydrogen isotopic compositions of *n*-C_16_ and *n*-C_18_ alkanes from hadal surface sediments differ from that in the hadal water and the δ_2_H of the sediment (−79 to −93‰) within the isotopic composition of heterotrophic bacteria (4). This suggests that *n*-C_16_ and *n*-C_18_ in these sediments may be derived from heterotrophic microorganisms (4), but the enzymes involved in their synthesis have not been identified. The predominance of medium-chain (C_18–24_) *n*-alkanes with a clear even-carbon number preference in hadal sediments (Fig. S2, Table S1) clearly suggests a microbial origin. The capacity for *n*-alkane biosynthesis (C_14–33_) is well documented in diverse bacteria, including *Clostridium pasteurianum*, *Bacillus* spp., and members of the Actinobacteria (including *Arthrobacter* sp., *Corynebacterium* sp., *Mycobacterium* sp. and *Micrococcus* sp.) (70). Here, we identified members of the phyla *Planctomycetota*, *Verrucomicrobiota*, *Actinobacteriota,* and *Proteobacteria* that contain putative olefin-synthesis genes (*olcBC*), suggesting they may be candidates for hydrocarbon production in hadal sediments (Table S11) (15), thereby supporting our hypothesis of *in situ* hydrocarbon synthesis.

Based on our findings, we propose a comprehensive framework for hydrocarbon metabolism in the hadal sediments (Fig. 6). According to this framework, subsurface hydrocarbons are likely sourced both by deposition from the water column and *in situ* microbial biosynthesis. Once deposited in the hadal sediments, these hydrocarbons are degraded through diverse aerobic and anaerobic pathways mediated by a metabolically versatile microbial community. This process couples hydrocarbon turnover with the biogeochemical cycles of carbon, nitrogen, and sulfur in the deep biosphere.

### Conclusion

This study reveals the highly stratified hydrocarbon metabolic potential of the microbial community inhabiting the deepest sediments (at a depth of 10,816 m) of the Challenger Deep in the Mariana Trench. Hydrocarbons and their associated degradation genes are abundant in subseafloor sediments, particularly within the uppermost 100 cmbsf of the sediment core. The distribution of *n*-alkane degradation genes corresponds to the concentration of *n*-alkanes in the hadal sediment core, suggesting potential hydrocarbon cycling. Bacterial and archaeal groups with relatively high abundances in the sediments, such as Chloroflexota, Pseudomonadota, Planctomycetota, Actinomycetota, Bacteroidota, Nitrospinae, Firmicutes, and Myxococcota, were identified as key potential primary hydrocarbon degraders. The microbial community in the sediment differs from that in the overlying waters, suggesting distinct metabolic adaptations. Moreover, the detection of potential alkane-synthesizing microorganisms suggests a possible *in situ* source of *n*-alkanes within the sediments. Together, these observations provide new insights into the potential hydrocarbon transformation processes and the ecological roles of microbial groups in the subseafloor carbon cycle of the deepest hadal sediments.

## MATERIALS AND METHODS

### Sample collection

A continuous sediment core (~7.5 m in length) was collected from the Challenger Deep of the Mariana Trench (11°19.904′N, 142°12.083′E, depth: 10,816 m) aboard the R/V *Dong Fang Hong 3* in July 2020 (Fig. S1) (2, 71). The core was sliced and subsampled using disposable sterile spatulas. To avoid contamination, the outer 1 cm of the sediment was sliced off, and the uncontaminated center of the remaining core sample was then subdivided for multiple analyses: 45 subsamples for *n*-alkane extraction (Table S1), 22 subsamples transferred to sterilized plastic tubes for DNA extraction (stored at −80°C, Table S2), and 23 subsamples for microbial cultivation (stored at 4°C, Table S3).

### Determination of *n*-alkane concentration in sediments

The 45 subsamples for alkane content determination (Table S1) were placed in tin foil immediately after subsampling and were promptly analyzed for hydrocarbon content. The measured method was following the one described by Liu et al. (4). Briefly, 5 g of the sediment sample was extracted with 30 mL dichloromethane/methanol (9:1, vol/vol) for 10 min. This procedure was repeated four times, and the extracts were combined. A blank control was performed using dichloromethane and hexane. Sediment extraction was conducted in a clean room, and glassware was baked at 550°C for 12 h to eliminate any organic contaminants. *n*-alkanes were analyzed using an Agilent 7890B gas chromatography (GC) with a flame ionization detector (FID). *n*-Alkanes were separated using a HP-1 column (50 m × 0.32 mm × 0.17 μm) programmed from 50°C to 300°C at a rate of 3°C /min and held at 300°C for 30 min. Helium was used as a carrier gas, with a flow rate set to 1 mL/min. Individual *n*-alkanes were identified by comparing retention times with those of *n*-alkane standards (Sigma), and their concentrations were calculated using standard calibration curves for each compound. The recovery efficiency for the internal standard was 92 ± 5%, so no correction was applied for standard recovery. This method predominantly targets nonvolatile alkanes (≥C_18_). Consequently, it may underestimate the concentrations of shorter-chain lengths (fewer than 18 carbon atoms) as their loss is likely due to methodological limitations in retaining volatile compounds during extraction.

To determine *n*-alkane sources, we analyzed *n*-alkane average chain length (ACL) and carbon preference index (CPI) (72, 73). Long-chain *n*-alkanes (*n* ≥ C_25_) are predominantly derived from terrestrial higher plants, such leaf waxes and grasses (72–74), while short- and mid-chain *n*-alkanes (*n* < C_25_) are mainly produced by bacteria and algae (72). The ACL value was calculated for *n*-alkanes with carbon number 18–36 according to the equation, where “*i*” is the carbon number (ACL$=\frac{\sum i\cdot Ci}{\sum Ci}$) (the ACL > 27 implies a potential contribution of terrigenous alkanes at these depths; otherwise, marine alkanes are considered dominant) (72, 73). The carbon preference index (CPI) is an indicator of organic matter (OM) maturity (72). The ACL value was calculated as follows:

$CPI_{\left(total\;n\text{-}alkane\right)}=\left(\left(\frac{\sum (C_{19}-C_{35})odd}{\sum (C_{18}-34)even})+(\frac{\sum (C_{19}-C_{35})odd}{\sum (C_{20}-36)even}\right)\right)/2$;

$CPI_{\left(mid\text{-}chain\;n\text{-}alkane\right)}=\left(\left(\frac{\sum (C_{19}-C_{23})odd}{\sum (C_{18}-C_{22})even})+(\frac{\sum (C_{19}-C_{23})odd}{\sum (C_{20}-C_{24})even}\right)\right)/2$;

$CPI_{\left(long\text{-}chain\;n\text{-}alkane\right)}=\left(\left(\frac{\sum (C_{25}-C_{35})odd}{\sum (C_{26}-C_{34})even})+(\frac{\sum (C_{25}-C_{35})odd}{\sum (C_{28}-C_{36})even}\right)\right)/2$.

Generally, CPI > 1.2 indicates immaturity, but CPI < 1.2 does not necessarily indicate maturity (74). CPI values of fungi, algae, and bacteria (CPI value ≤1.1) were reported obviously lower than those of terrestrial higher plants (74–76).

### Nucleic acid extraction and metagenomic sequencing

Total DNA was extracted from 10 to 12 g of sediment samples for each experimental replicate, as previously described (4, 71, 77). Twenty DNA samples (0–542 cmbsf, each about 1 μg) were sent to BGI TECH SOLUTIONS (BEIJING LIUHE) CO. LIMITED, and two DNA samples (649–652 cmbsf, 0.4 μg and 749–752 cmbsf, 0.2 μg) were sent to Majorbio BiFo-Pharm Technology Co. Ltd. (Shanghai, China) for metagenomic sequencing. Libraries were prepared without any amplification step for each sample. Metagenomic shotgun sequencing was performed on the Illumina HiSeq X-Ten platform, with 2 × 150 bp paired-end reads.

### Metagenomic assembly, mapping, and binning

Metagenomic assembly, mapping, and binning were performed according to Xue et al. (78) with modifications. Briefly, raw sequence data with >10% undefined bases, >40% low-quality bases, and >15 bases matching the adapters were removed with the metaWRAP-Read_qc module (79). The clean reads were then assembled with MetaSpades version v3.15.2 (80). Genome binning and refinement were performed on contigs (1.62 to 3.65 Gb) using the metaWRAP-binning module, a combination of three metagenomics binning software, MaxBin2 version 2.2.4 (81), metaBAT2 version 2.12.1 (82), and CONCOCT version 0.4.0 (83). Raw genome bins resulting from these three binning approaches were combined, and the best binning result was selected for each genome set using the bin_refinement module in metaWRAP with options: -c 50 -× 10. The completion and contamination of each bin were evaluated by CheckM version 1.0.7 (84). Good-quality metagenomic assembled genomes (MAGs; ≥50% completeness and ≤10% contamination) were retained for further analysis. Finally, a total of 1,766 MAGs were assembled. To simplify the analysis, we used dRep (85) (options: -comp, 75; -con, 25; -sa S_ANI, 0.99) to dereplicate 1,766 MAGs and obtained 342 medium-quality (≥75% completeness and ≤10% contamination) and high-quality (≥90% completeness and ≤5% contamination) dereplicated MAGs for further analysis.

The relative abundance of the 342 dereplicated MAGs was predicted using CoverM v0.3.1 (https://github.com/wwood/CoverM) with the “contig” command and a cutoff of 95% minimum identity and minimum aligned read length of 75% of each read. The coverage of each contig was calculated with the CoverM “trimmed_mean” option, and the coverage for each MAG was calculated as the average of all contig coverages, weighted by their length. The relative abundance of MAGs in each metagenomic data set was calculated as its coverage divided by the total coverage of all genomes in the dereplicated MAG data set.

### Phylogenetic analysis and taxonomy assignments of the metagenome-assembled genomes (MAGs)

The taxonomy of each MAG was obtained using GTDB-Tk (v2.3.2) (86) with the Genome Taxonomy Database (GTDB). A total of 120 bacterial and 122 archaeal marker genes from each MAG were used to build maximum likelihood (ML) phylogenomic trees using IQ-TREE version 1.6.1 (87) with the LG+R10 and LG+R4 model. For better visualization, the trees were constructed using the Chiplot web tool (https://www.chiplot.online/) (88).

### Annotated hydrocarbon-degrading-related genes in metagenomes and MAGs

Gene sequences were obtained using Prodigal version 2.6.3 (89) in default (“-p meta” for metagenome sequences) from the contigs of these 22 metagenome samples. Gene sequences were clustered into 77,864,662 nonredundant sequences using Cd-hit (90) with a similarity of 95% identity and 90% coverage.

CANT-HYD, a database of 37 hidden Markov models (HMMs) of marker genes involved in anaerobic and aerobic degradation pathways of aliphatic and aromatic hydrocarbons (23), was used to analyze the metagenomics data and 342 dereplicated MAGs with an e-value cutoff of 10^−50^. For each group of HYD genes annotated using CANT-HYD, we double-checked using BLASTp with an e-value cutoff of 10^−50^ against the reference proteins in the CANT-HYD database. An identity value of above 30% was selected as the target for HYD genes. The relative abundance of each gene category at each depth was calculated as the reads per kilobase of transcript per million mapped reads (RPKM) value (the RPKM value of gene homologs × 100)/(the RPKM value of *recA*). RPKM was confirmed using BWA-MEM (bwa version 0.7.17-r1188, MA, USA, using default setting) and samtools version 1.10 (71, 91). The figures were drawn using software Origin v 2024.

### Annotated potential hydrocarbon synthesis-related genes in MAGs

Annotation of potential hydrocarbon synthesis-related genes in MAGs was performed according to Vigneron et al. (15). All of the 342 dereplicated MAGs were annotated using KEGG annotation (https://www.kegg.jp/ghostkoala/, genus_prokaryotes + family_eukaryotes +viruses) and then searched for key hydrocarbon synthesis genes, including algal fatty acid photodecarboxylase (FAP; K22464), squalene synthase (SSL; K21149 and K21149), fatty aldehyde decarbonylase (FAD; K14331), aldehyde deformylating oxygenase (ADO; K14331), and olefin beta-lactone synthetase (OleBC and OleC, K25044 and K22319).

### Quantitative PCR of *alkB* in different sediment layers

The abundance of *alkB* genes was quantified based on the methods of Liu et al. (4). The linear plasmid was purified using a Gel Extraction Kit (Omega) and was serially diluted to construct standard curves (concentration ranged from 3.49 × 10^1^ to 3.49 × 10^8^ copies/L). Each sample was run with negative controls conducted in triplicate performed on the QuantStudio 5 system (Thermo Fisher Scientific) based on TB Green Premix Ex Taq (Tli RNaseH Plus) (Takara). Each 20 μL reaction mixture contained 10 μL TB Green Premix Ex Taq, 0.4 μL ROX Reference Dye (50×), 10 μM (0.8 μL) of each forward and reverse primers, 2 μL of diluted (1:5) DNA template, and 6 μL nuclease-free water. The qPCR cycling parameters involved an initial activation step at 95°C for 120 s, followed by 40 cycles of a 2-step reaction involving denaturation at 95°C for 20 s, annealing at 52°C for 20 s, and extension at 72°C for 40 s. To confirm that each primer pair produced only a single specific product, a melting curve was added to the end of every qPCR assay at every run (for all primers).

### Characterizing the alkane-degrading ability of AlmA in Chloroflexota

Characterization of the *alma* gene in the Chloroflexota M49-52cm_MAG58 genome (order: UBA2979, phylum: Chloroflexota) was performed according to the method described by Wang et al. (92). Construction of the recombinant expression plasmid pRSET-A-*almA* is shown in Fig. S9a. Briefly, *almA* (MDBPCMKO_01555) along with the genes encoding two required electron transport proteins, MDBPCMKO_00267 (ferredoxin reductase 2) and MDBPCMKO_00787 (ferredoxin), were inserted into the expression plasmid pRSET-A. A promoter and ribosome-binding site (RBS) were included upstream of both the electron transport protein-encoding genes and *almA*. The recombinant plasmid was constructed by Universe Gene Technology (Tianjin) Co., Ltd. To confirm the successful insertion of *almA*, specific primers of the *almA* gene (MDBPCMKO_01555) were designed (*almA*-432-F: 5′-CGCCAATTTCCTCTGGATGTGCCAG-3′; *almA*-1120-R: 5′-GCTTGCCGTCGATCTCGAAGTCGAT-3′). PCR amplification of the recombinant plasmid was carried out using *TaKaRa Ex Taq* (TaKaRa, Japan), according to the manufacturer’s instructions, with an annealing temperature of 55°C.

The recombinant plasmid pRSET-A*-FF-almA* and pRSET-A (10 μL) was transformed into *E. coli* BL21 (Bomaide Biological, Beijing) and selected on LB solid plates containing ampicillin (final concentration: 50 μg/mL) and chloramphenicol (35 μg/mL). Single colonies were picked and transferred to 5 mL of fresh LB liquid medium containing ampicillin (final concentration: 100 μg/mL) to prepare seed cultures. Two milliliters of seed culture was inoculated into 150 mL of LB liquid medium containing antibiotics and incubated at 37°C, 170 rpm, until the OD_600_nm reached 0.5–0.6. Isopropyl β-D-thiogalactoside (IPTG) was then added to a final concentration of 1 mM, and the culture was returned to the shaker for an additional 2 h to induce plasmid expression.

Alkane degradation activity was measured by washing induced cells with M9 medium containing glucose and antibiotics (M9-glucose), followed by resuspension in 90 mL of fresh M9-glucose medium containing antibiotics. One milliliter of the resuspended cells was added to 10 mL of mixed alkanes (C_18_ and C_19_, each at a concentration of 2.5 mg/mL) in ORN7a medium. The culture was then incubated at 37°C, 170 rpm for 80 h. Two additional control groups, *E. coli* BL21 and *E. coli* BL21-pRSET-A, were included, and an uninoculated blank medium was used as a negative control. Each group had three replicates. Finally, the remaining alkane content in the culture was extracted to assess the alkane degradation ability of the recombinant strain. One hundred microliters of the internal standard (deuterated-hexacosane: 400 μg) was then added to the culture medium, followed by the addition of 10 mL of dichloromethane (DCM). The mixture was gently shaken and then placed in an ultrasonic bath (40 Hz) for ultrasonic extraction for 10 min. After sonication, the lower organic phase of the culture was carefully transferred to a new 25 mL glass bottle. Next, 5 mL of DCM was added to the culture medium, and ultrasonic extraction was performed for 5 min. The organic phase was then transferred to the same 25 mL bottle. This extraction was repeated once more, yielding approximately 20 mL of the extract. Anhydrous sodium sulfate, previously heated in a muffle furnace to remove carbon, was added to the extract to remove any remaining water. One milliliter of the processed extract was transferred to a 2 mL GC vial for analysis.

The alkane content in the extract was detected using an Agilent 8860GC System and HP-5 (30 m × 320 μm × 0.25 μm) chromatography column, with helium as the carrier gas at a flow rate of 1.9775 mL/min. The temperature program was as follows: initial temperature of 60°C, held for 1 min, then raised at 5°C/min to 280°C, and held for 25 min. The detector used was a flame ionization detector (FID). Two microliters of the sample was injected using a 10 μL syringe. The alkane types present in the sample were identified based on the retention time of standards (Sigma), and the concentration of each alkane in the sample was calculated using a standard curve. All glassware used in the above processes was cleaned by combustion at 450°C for 6 h to remove organic contamination.

### Three-dimensional structure prediction and catalytic active site analysis of the AhyA proteins

Two previously reported AhyA proteins (NCBI accession numbers: WP_012173623 and Oqx63933) were selected as references to predict the three-dimensional structures, catalytic sites, and alkane-binding affinities of two AhyA proteins (AhyA_120_123_587 and AhyA_120_123_580) encoded by Chloroflexota MAGs (M120-123cm_MAG43). To prepare the small-molecule ligands, the structural formulas of C_10_ and C_20_ were generated using an online tool (https://mole.chemview.net/?cid=15600) and exported as MOL files. Each MOL file was then saved as a PDB file using PyMOL v3.1 (93) to obtain the three-dimensional structures required for docking. For protein structure prediction, the AhyA sequences were submitted to the Alphafold3 Server (https://alphafoldserver.com/) (default parameters). The resulting PDB files were subsequently used for molecular docking with Autodock v1.5.7 (94) to predict binding energies between the enzymes and ligands and to identify catalytic active sites. The predicted protein structures and docking sites were visualized and polished using PyMOL v3.1.

### Enrichment of potential alkane-degrading bacteria and evaluation of alkane-degrading ability of representative strains

Sediment samples from 23 layers (Table S3) were enriched using 50 mL ORN7a medium supplemented with 1% mixed *n*-alkanes (C_6_, C_11_, C_16_, C_19_, C_22_, C_25_, C_28,_ and C_32_) or a petroleum:diesel (1:4 [M:M]) mixture as the sole carbon source. Cultures were incubated at 16°C and atmospheric pressure for 20 days (medium composition in Table S3) (Fig. S16a). The medium was diluted to an appropriate concentration (10⁻³ or 10⁻⁴), depending on colony density in the enrichment system. Then, 100 μL of the diluted culture was spread onto an ORN7a agar plate, followed by the addition of 60 μL of mixed *n*-alkanes or petroleum : diesel to evenly coat the plate and incubated at 16°C for 7 days. When distinct morphological colonies were visible, individual colonies were randomly selected, purified by streaking three times on fresh media, and incubated at the same temperature. Following identification by 16S rRNA gene sequencing, stocks were preserved at −80°C in 0.85% (wt/vol) NaCl supplemented with 15% (vol/vol) glycerol (95). Taxonomic assignment was determined using the EzBioCloud server (http://www.ezbiocloud.net/).

Incubation of individual colonies was performed in 10 mL of ORN7a medium supplemented with *n*-alkane with different chain lengths (C_18_, C_19_, C_20_, C_21_, C_22_, C_24_, C_25_, C_27_, C_28_, C_31_, C_32_, and C_36_, 500 μg each) at 5°C and atmospheric pressure (0.1 Mpa) for 30 days. After confirmation that these species degraded alkanes at low temperature, we further assessed their capacity to degrade *n*-alkanes under high-pressure and low-temperature conditions. Strains were incubated in 5 mL of ORN7a medium supplemented with 2 mg *n*-octadecane at 20, 40, and 60 MPa for 20 days. Their hydrocarbon degradation ability under high pressure and low temperature (maximum pressure tolerance) was evaluated over 30 days at 5°C. High-pressure incubations were conducted in stainless steel reactors (380 mL, maximum pressure 60 MPa; Nantong Feiyu Oil Science and Technology Exploitation, China), with pressure applied by a manual water pump. After the incubation period, the remaining alkanes were extracted immediately using dichloromethane and analyzed using an Agilent 7890B gas chromatography (GC) equipped with an FID detector.

### Enrichment culture of potential alkane-synthesizing bacteria and evaluation of alkane-synthesizing ability of representative strains

Sediment samples from 23 layers (Table S3) were enriched in 50 mL of ORN7a medium containing 0.6% sodium pyruvate as the sole carbon source and incubated at 28°C and atmospheric pressure for 7 days. The medium was diluted to an appropriate concentration (10⁻³ or 10⁻⁴) depending on colony density in the enrichment system. One hundred microliters of the diluted culture was spread on ORN7a agar plates with 0.6% sodium pyruvate as the sole carbon source and incubated at 28°C for 7 days. When distinct morphologically different colonies appeared, individual colonies were randomly selected, purified by streaking three times on fresh media, and incubated at the same temperature. Taxonomic assignment was determined according to the methods described above (95).

Gram-positive aerobic bacteria, such as *Bacillus* sp., *Arthrobacter* sp., and *Micrococcus* sp., can reportedly synthesize C_14–34_, C_15–34_, and C_17–30_ *n*-alkanes, respectively (70, 75). Three representative strains, *Bacillus subtilis* HXX016, *Arthrobacter humicola* HXX366, and *Oceanobacillus kimchi* HXX416 (as a negative control), isolated in this study were selected to test their alkane-synthesizing ability. Incubations were carried out in 10 mL of ORN7a medium supplemented with 0.6% sodium pyruvate, 1/2 marine broth (MB; per liter of seawater: 2.5 g peptone, 0.5 g yeast extract, and 0.05 g FePO₄) 0.3% sodium pyruvate, and 1/2 MB with 0.3% sodium pyruvate. Cultures were incubated at 28°C for 7 days. After incubation, the culture was immediately extracted with 20 mL dichloromethane, and the presence of alkanes was analyzed using an Agilent 7890B Gas Chromatograph (GC) with an FID detector.

## Data Availability

The raw data for 22 metagenomes were submitted to the National Omics Data Encyclopedia under accession numbers OEP00003305, as well as submitted to the NCBI under accession number PRJNA957232. The raw data for 16S rRNA gene amplification sequencing were submitted to the National Omics Data Encyclopedia under accession number PRJCA008837. The 1,766 MAGs were deposited in eLMSG (an eLibrary of Microbial Systematics and Genomics, https://www.biosino.org/elmsg/index) under accession numbers LMSG_G000011653.1 to MSG_G000013418.1.

## SUPPLEMENTAL MATERIAL

File S1 mbio.03943-25-s0001.docx Fig. S1–S19 and captions for Tables S1–S13.

File S2 mbio.03943-25-s0002.xlsx Tables S1–S13.

ASM does not own the copyrights to Supplemental Material that may be linked to, or accessed through, an article. The authors have granted ASM a non-exclusive, world-wide license to publish the Supplemental Material files. Please contact the corresponding author directly for reuse.
