# Supplementary material for: Metabolically diverse microorganisms mediating hydrocarbon cycling in the subseafloor sediment of the Challenger Deep
Source: mBio. 2026 Apr 13;17(5):e03943-25. doi: 10.1128/mbio.03943-25 (PMC13170169; doi:10.1128/mbio.03943-25)
Supplement: File S1 — Fig. S1–S19 and captions for Tables S1–S13. [file mbio.03943-25-s0001.docx]

**
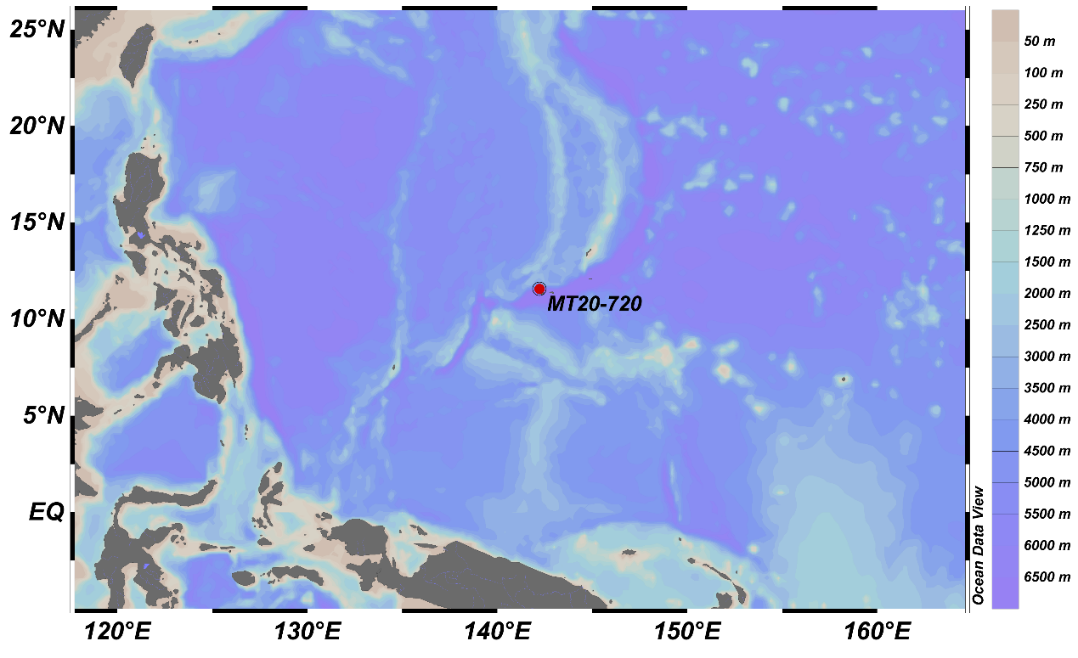
**

**Fig. S1 The Challenger Deep area of the Mariana Trench**. The intact sediment core sample (about 7.5m length) was collected from the Challenger Deep in the Mariana Trench (11°19.904’N, 142°12.083’E, depth: 10,816 mbsl).


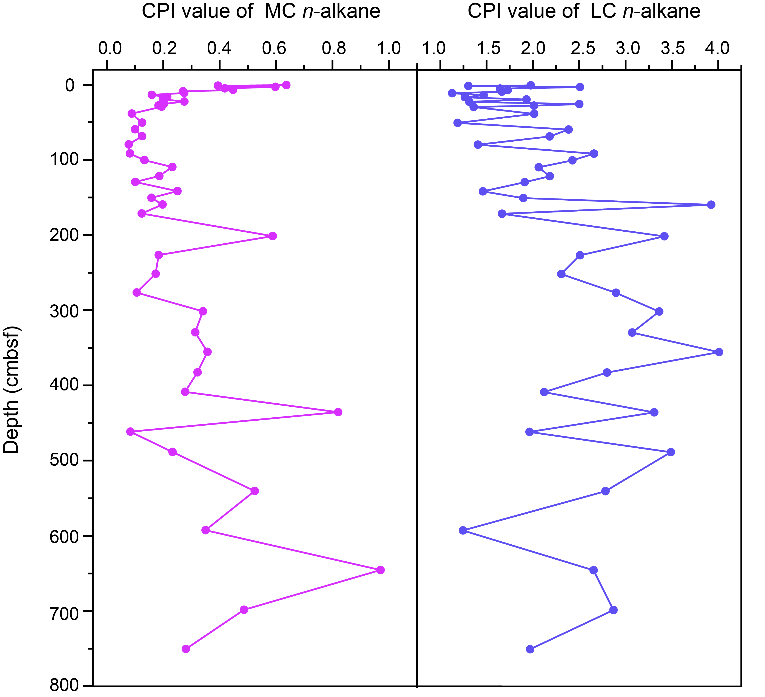


**Fig. S2 The CPI values of medium-chain (C_18-24_) and long-chain (C_25-36_) *n*-alkanes across the sediment core.**


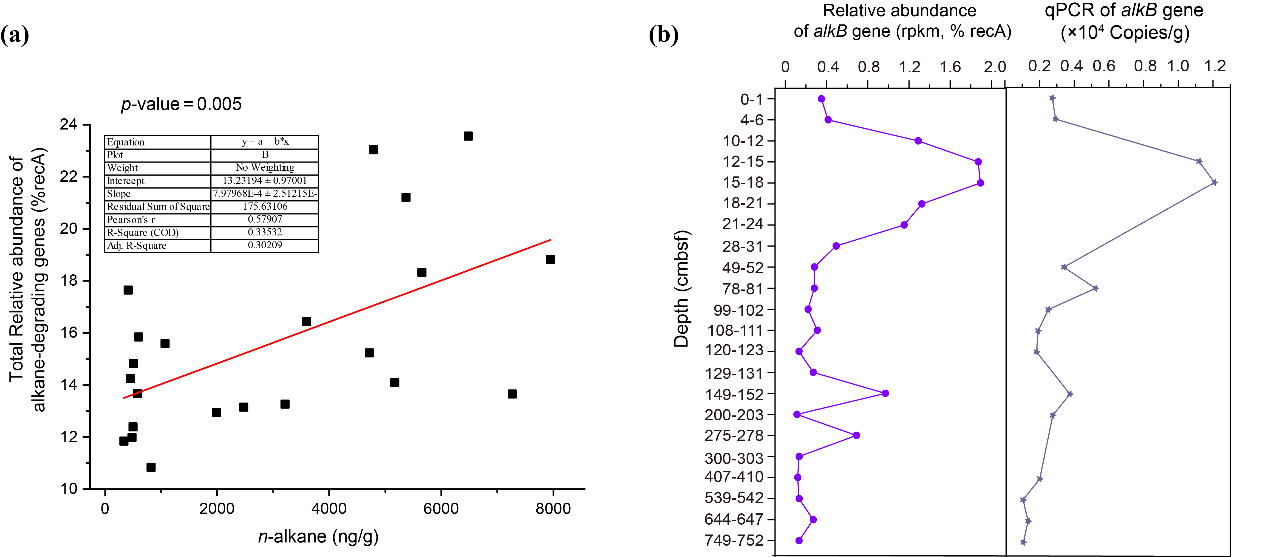


**Fig. S3** **The abundance of *n*-alkane degradation genes correlated to *n*-alkane content.** (a) Pearson's coefficient was used to calculate the correlation between the *n*-alkane content and total alkane degrading genes *in situ*. *p*-value=0.005. X-axis: Concentration of total n-alkanes *in situ*; Y axis: Relative abundance of alkane-degrading genes *in situ*. (b) The variation in the absolute abundance of the *alkB* gene (by qPCR) and its metagenomic relative abundance along the sediment depth gradient were strongly correlated (*p*-value <0.01, Spearman rank correlation analysis).
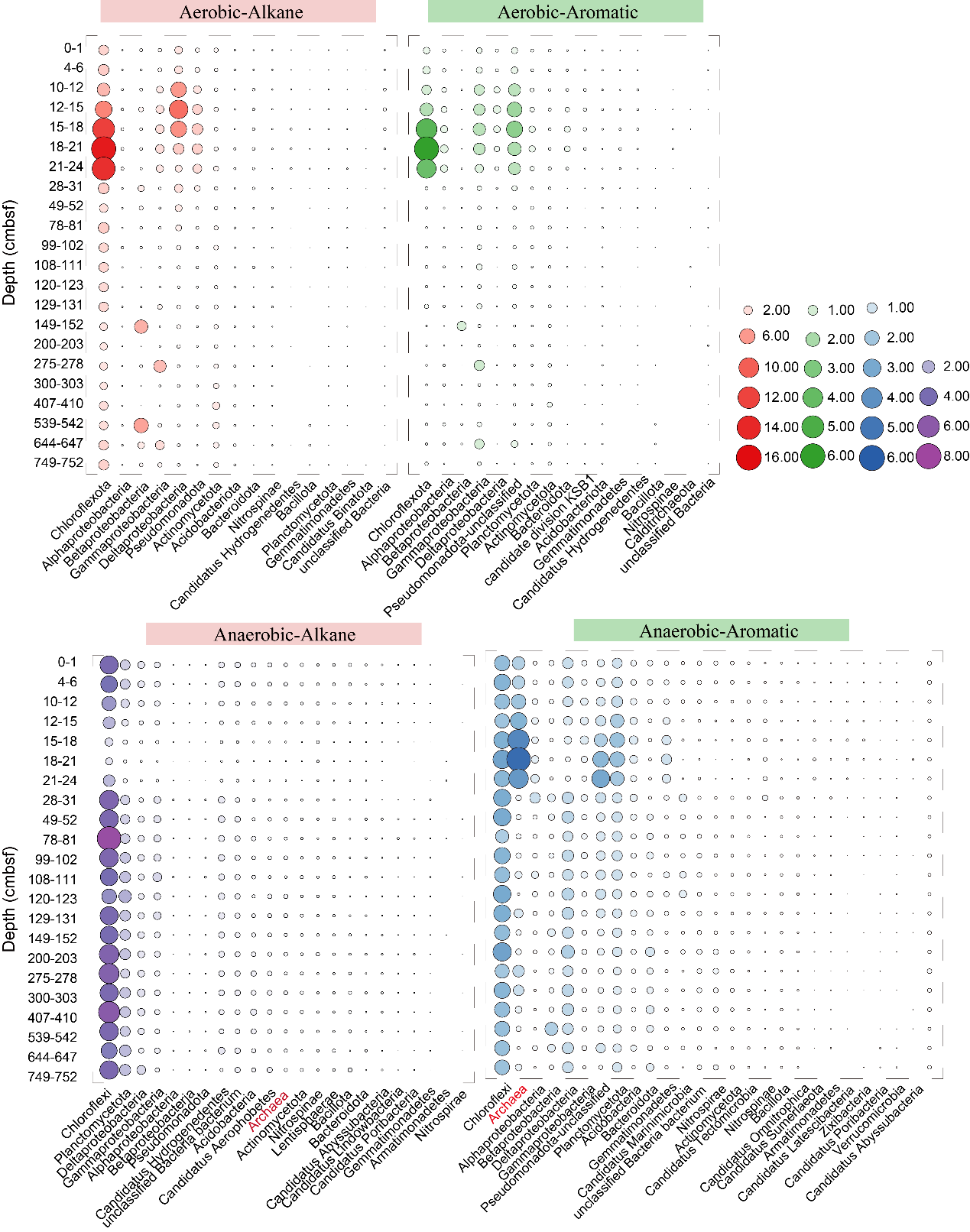


**Fig. S4 Depth distributions of four groups of hydrocarbon oxidizers (microbiomes which encode aerobic-alkane, aerobic-aromatic, anaerobic-alkane and anaerobic-aromatic** **degradation pathways).**


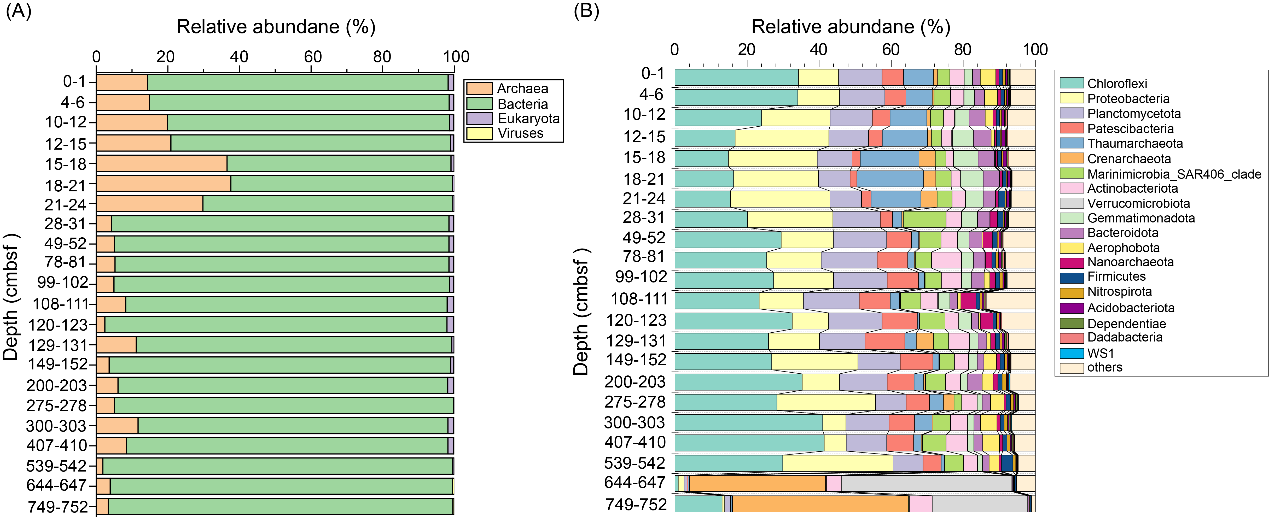


**Fig. S5 Microbial taxonomic profiles throughout the sediment samples in the Challenger Deep.** (a) the Kingdom level, (b) the phylum level. The analysis was performed by annotating the metagenomic data against the NCBI-nr database using v-search.
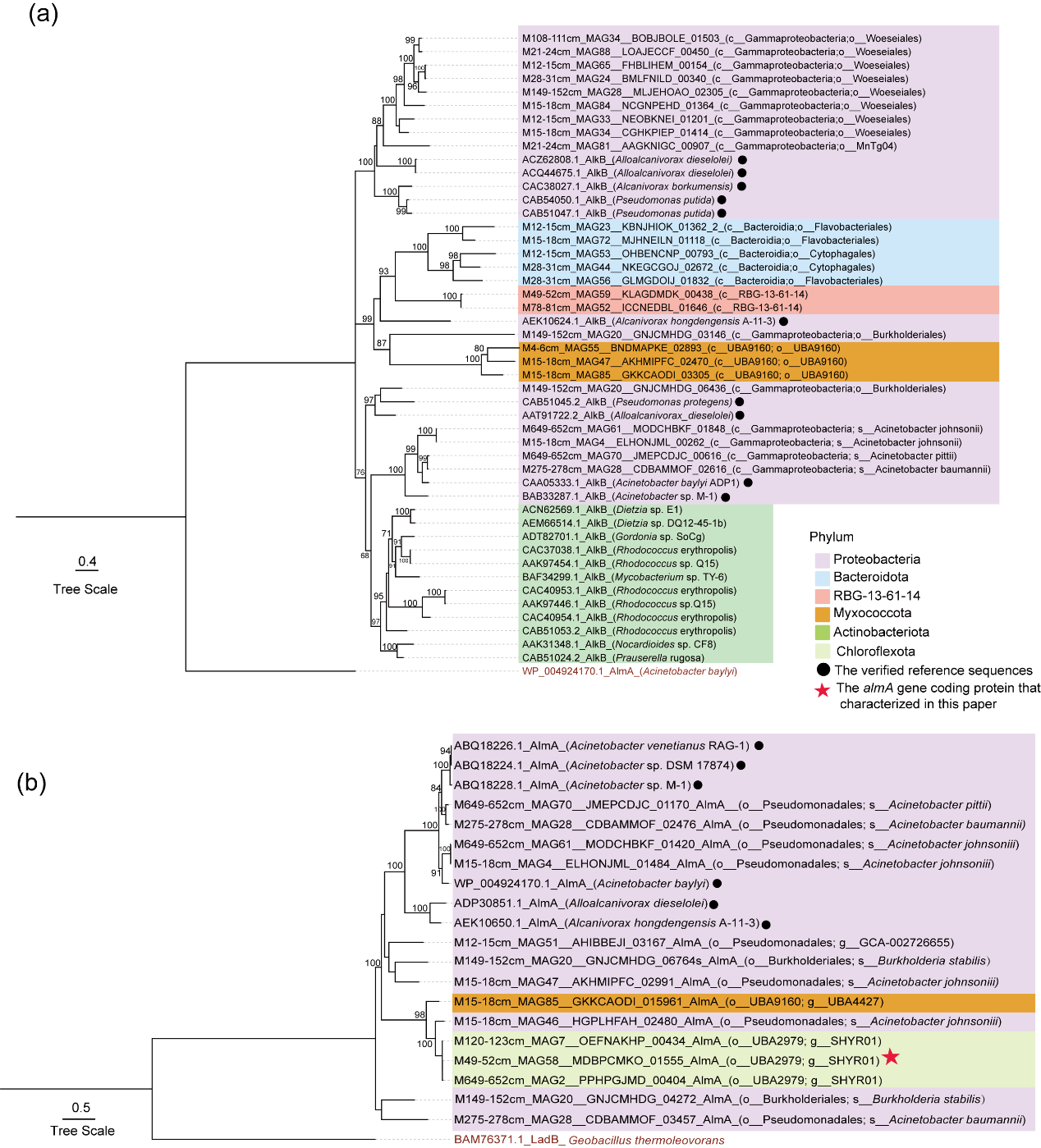

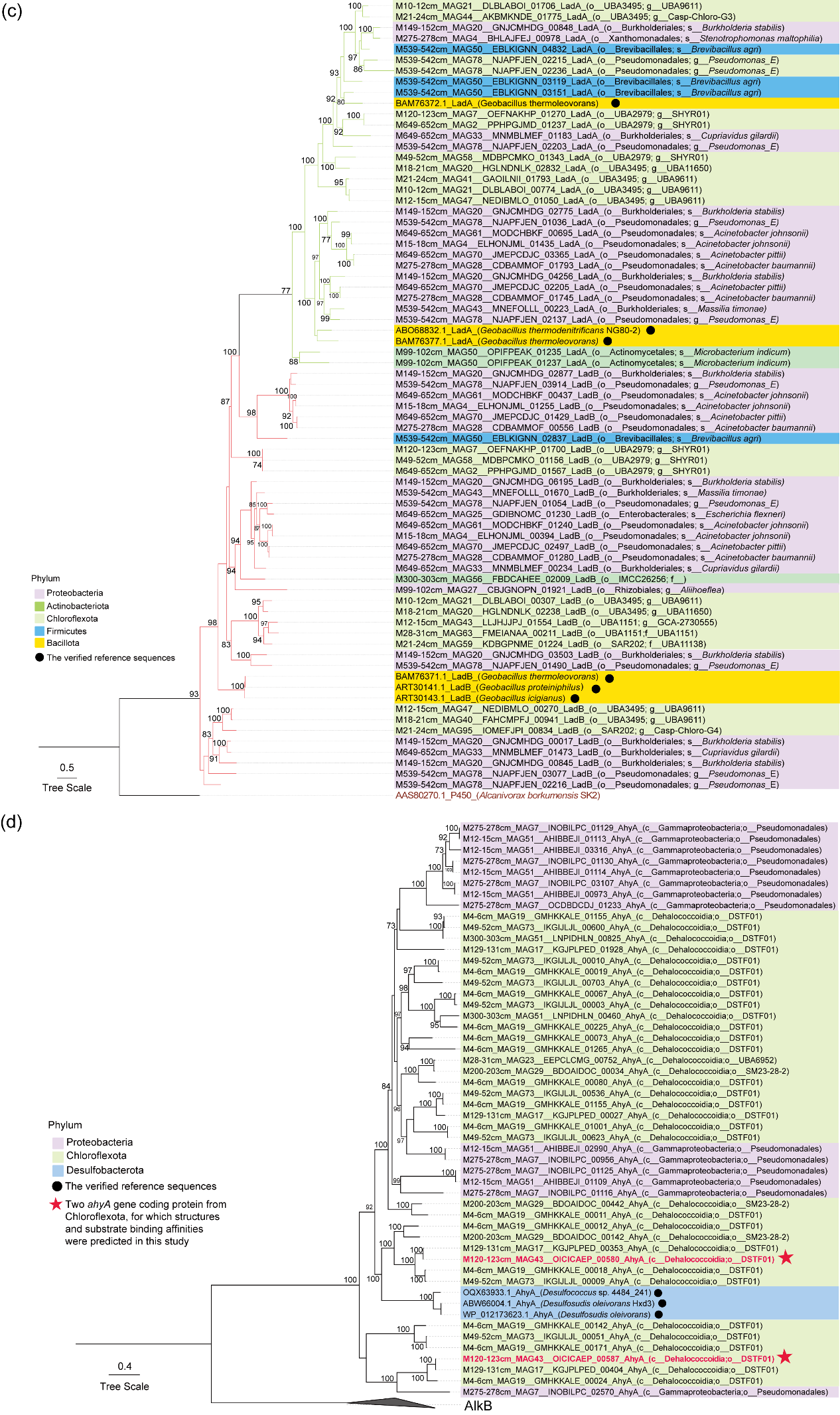


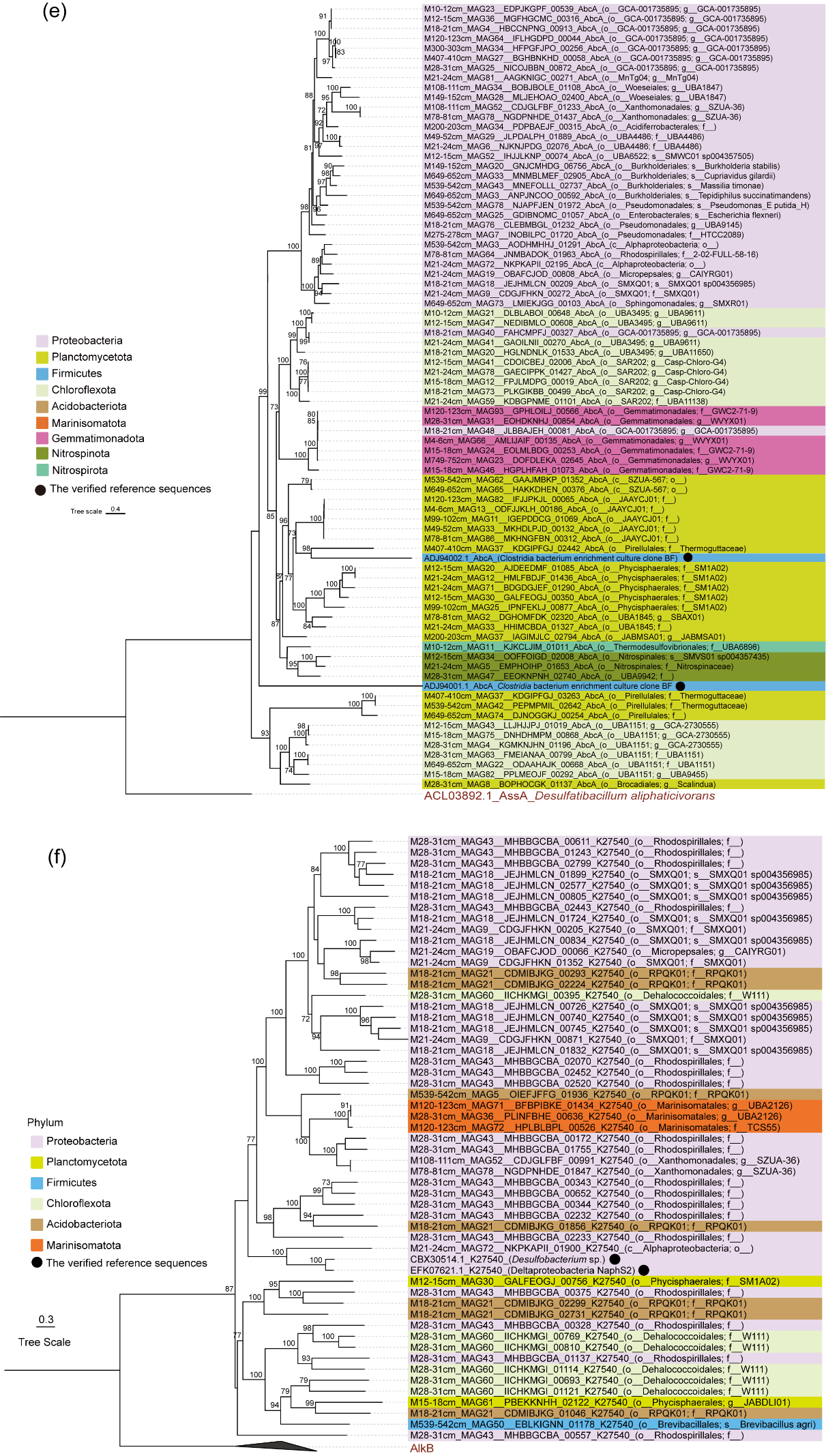


**Fig. S6 Maximum likelihood phylogenetic tree of HYD genes encoded by 120 HYD MAGs from hadal sediments.** Proteins encoded by (a) *alkB*, (b) *almA*, (c) *ladAB*, (d) *ahyA*, (e) *abcA*-like and (f) *k27540*-like homologs. Black dots denote reported reference genes, red pentagrams indicate the two proteins verified in this study. Different branch background colors indicate that the gene is encoded by different taxa.

**
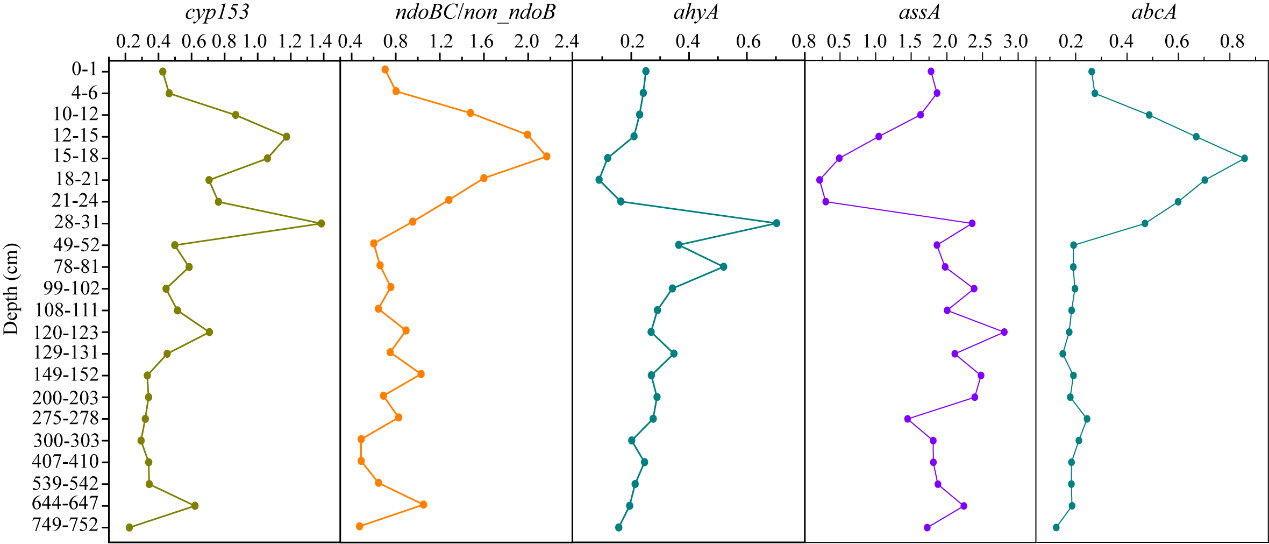
Fig. S7 Relative abundance of hydrocarbon-degrading MAGs harboring genes from five categories in the sediment core.**


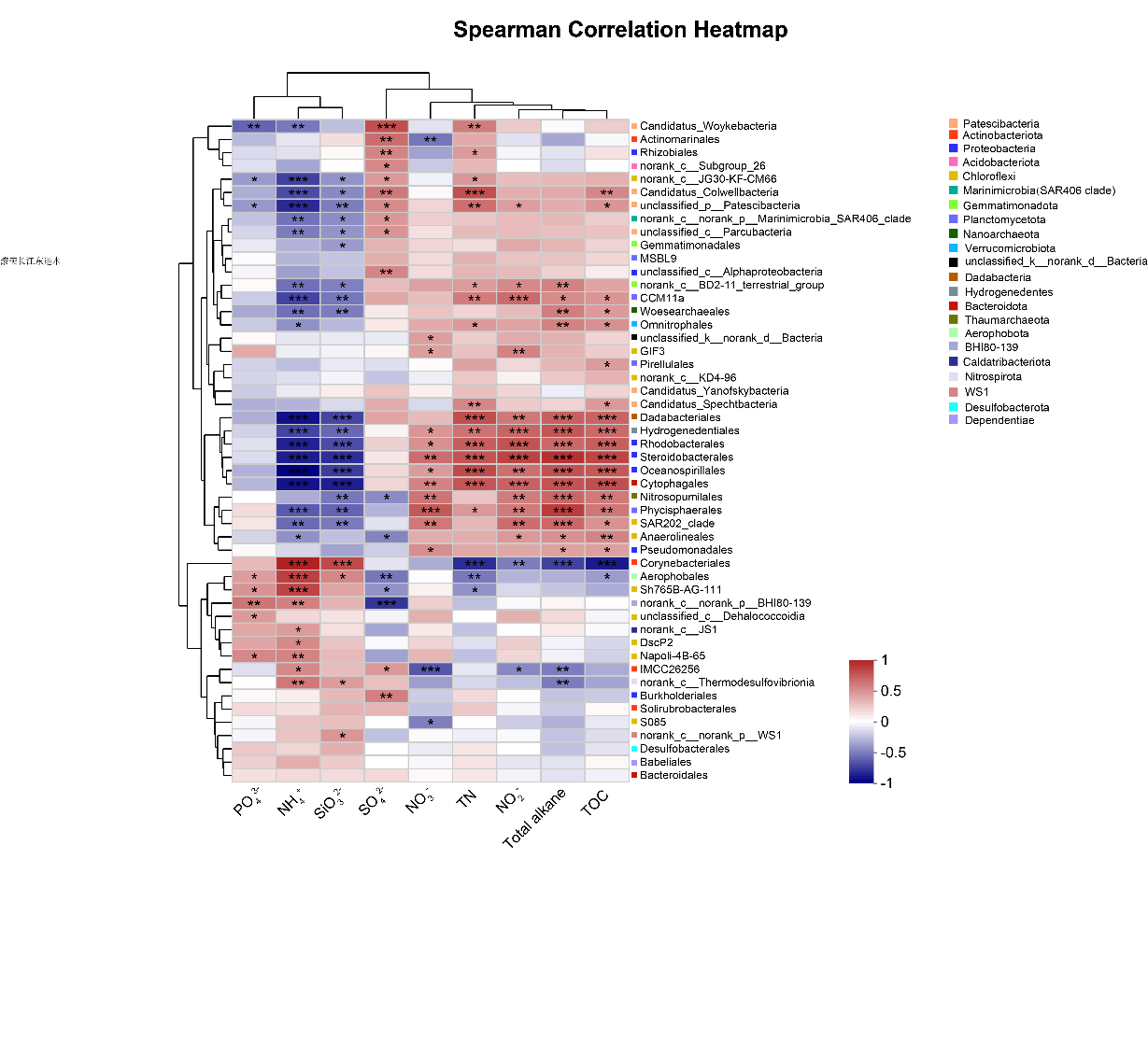


**Fig. S8** **Heat map showing the correlation between dominant orders (based on 16s rRNA sequencing) and environmental factors outlined in Liu et al. 2024** (1). ** indicates a *p*-value of <0.01 and *** indicates a *p*-value of <0.001.


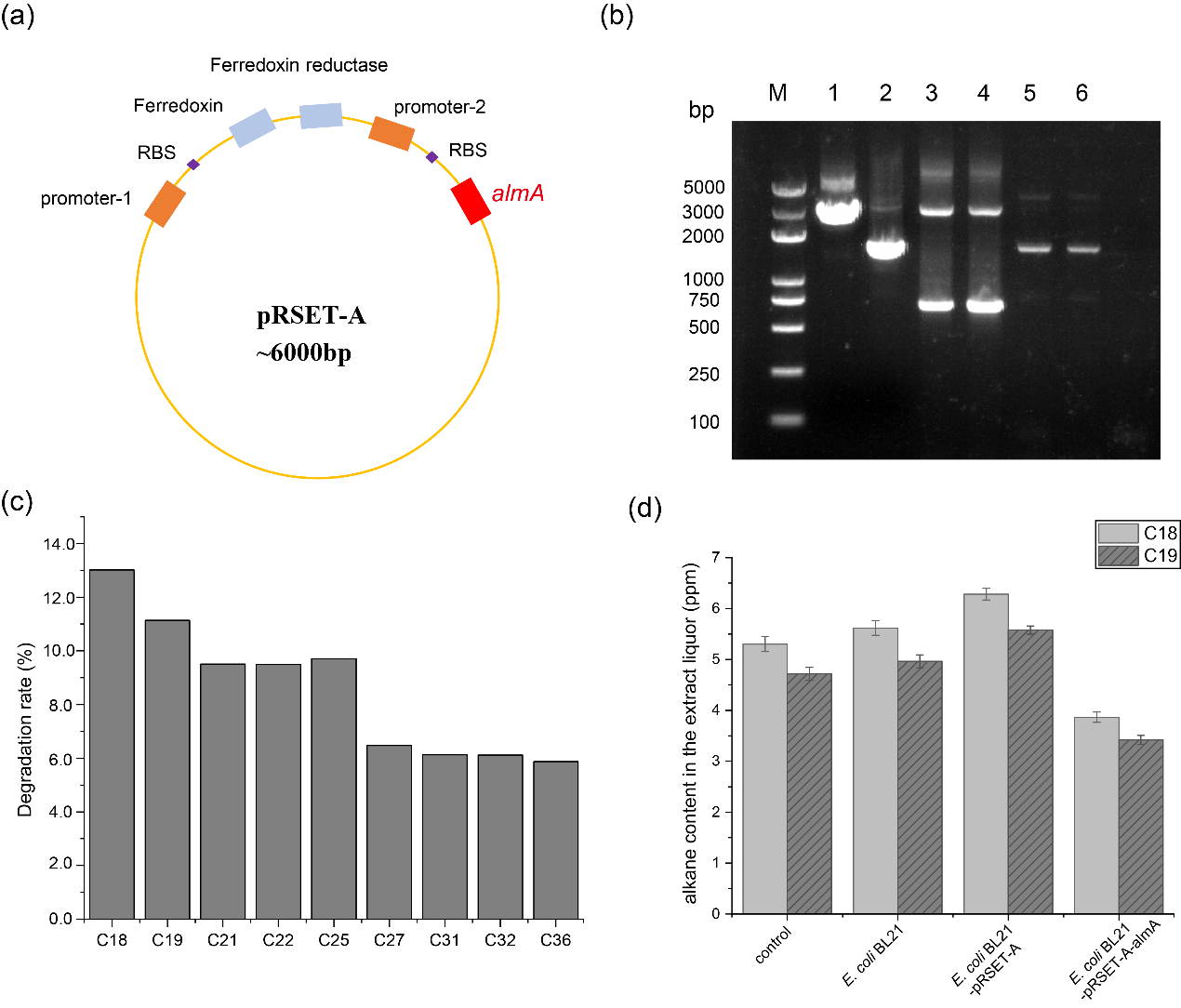


**Fig. S9 The cloning and expression of *almA* in M49-52cm_MAG58 from the order UBA2979.** (a) Construction of the expression vector (pRSET-A-FF-almA) was performed according to Wang et al. 2014 (2). To ensure the efficiency of expression, the ferredoxin and ferredoxin reductase genes (MDBPCMKO_00267 and MDBPCMKO_00787) were inserted upstream of *almA*, and two RBS gene were inserted before *almA* and the ferredoxin and ferredoxin reductase genes (FF). (b) M: marker; 1: recombinant plasmid pRSET-A-FF-*almA*; 2: plasmid pRSET-A; 3-4: plasmid pRSET-A-FF-*almA* using *almA* gene validation primers (*almA*-432-F: 5’-CGCCAATTTCCTCTGGATGTGCCAG-3’; *almA*-1120-R: 5’-GCTTGCCGTCGATCTCGAAGTCGAT-3’); 5-6: plasmid pRSET-A using *almA* gene validation primers. (c) *n*-alkane degradation ability for recombinant strain *E.coli* BL21-pRSET-A-FF-*almA* after 36 h. (d) *n*-alkane degradation ability for recombinant strain *E.coli* BL21-pRSET-A-FF-*almA* after 72 h.


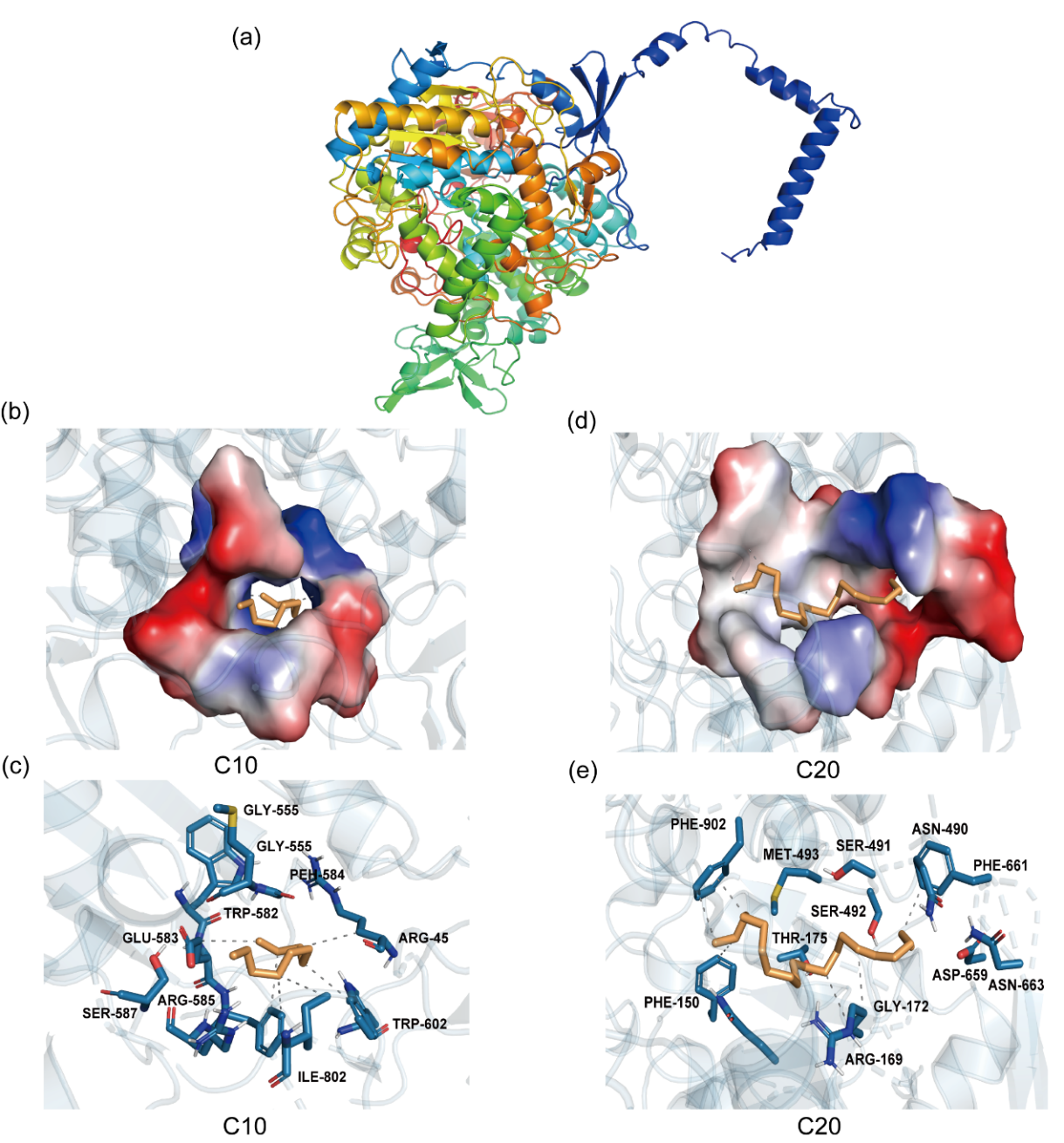


**Fig. S10 Prediction of the three-dimensional structure of the AhyA Protein (AhyA120_123_587) encoded by Chloroflexota based on AlphaFold3 and its catalytic hydrocarbon degradation binding sites with alkanes of different chain lengths (****C_10_ and C_20_).**  (a) Predicted three-dimensional structure of alkane hydroxylase AhyA120_123_587. (b) Molecular docking models illustrating its binding with C_10_ and C_20_). (c) Close-up views of the corresponding key substrate binding sites with C_10_. (d) Molecular docking models illustrating its binding with C_20_. (e) Close-up views of the corresponding key substrate binding sites with C_20_.


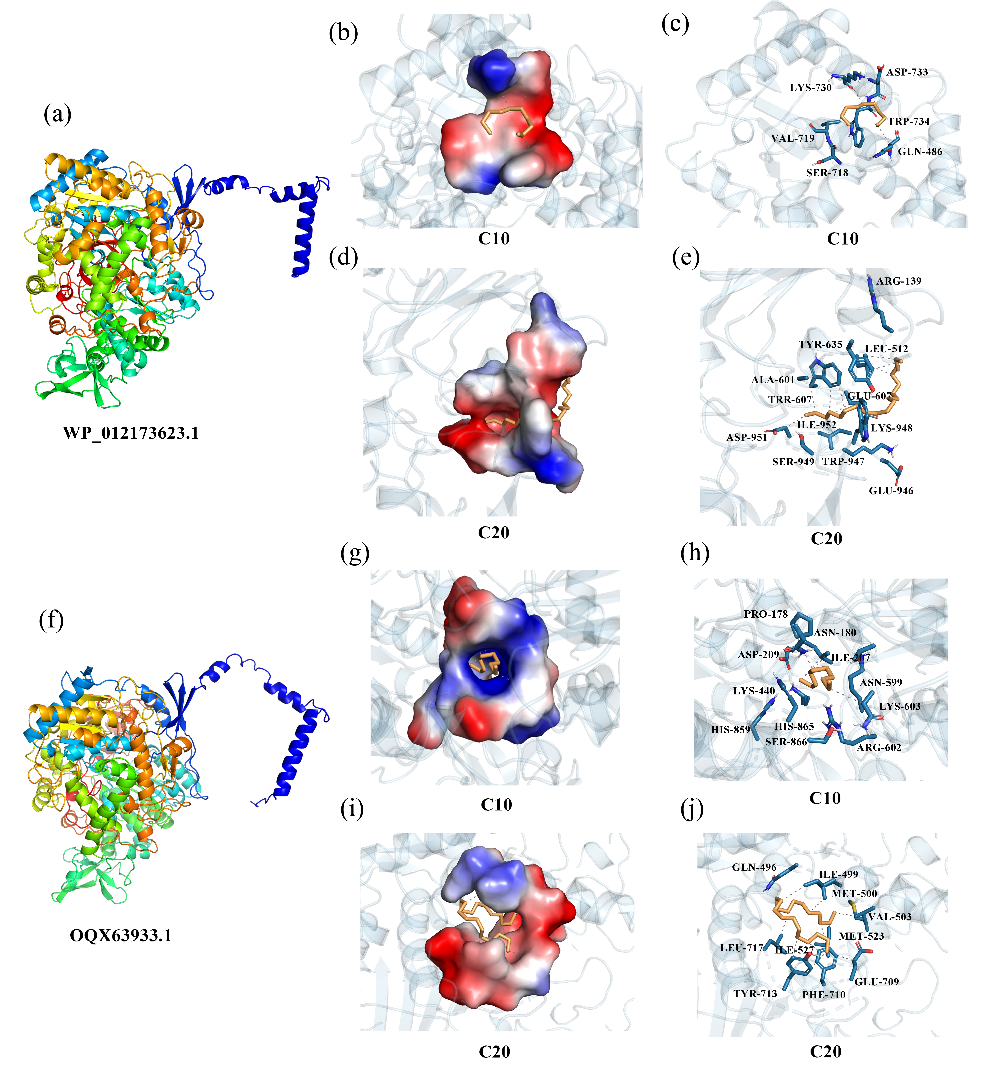


**Fig. S11 Three-dimensional structures and catalytic models of two reference anaerobic alkane hydroxylases (AhyA).** Predicted structure of WP_012173623 (a). Molecular docking models of WP_012173623 with alkanes of different chain lengths (C_10_ and C_20_) (b, d). Close-up views of the corresponding key substrate binding sites (c, e). Predicted structure of Oqx63933(f). Molecular docking models of Oqx63933 with C_10_ and C_20_ alkanes (g, i). Detailed views of its pivotal substrate binding residues (h, j).

**
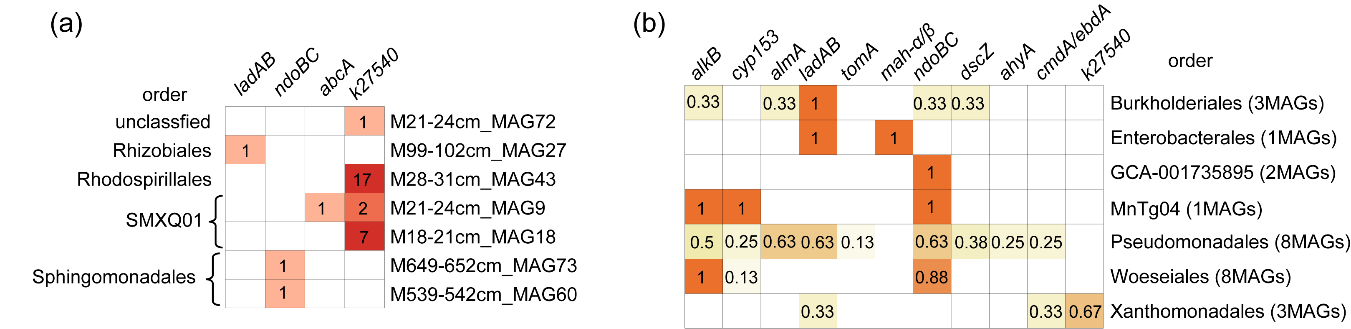
**

**Fig. S12** **Hydrocarbon-degrading genes encoded by the MAGs from *Proteobacteria* in the core.** The hydrocarbon degrading genes encoded by (a) Alphaproteobacteria and (b) Gammaproteobacteria.


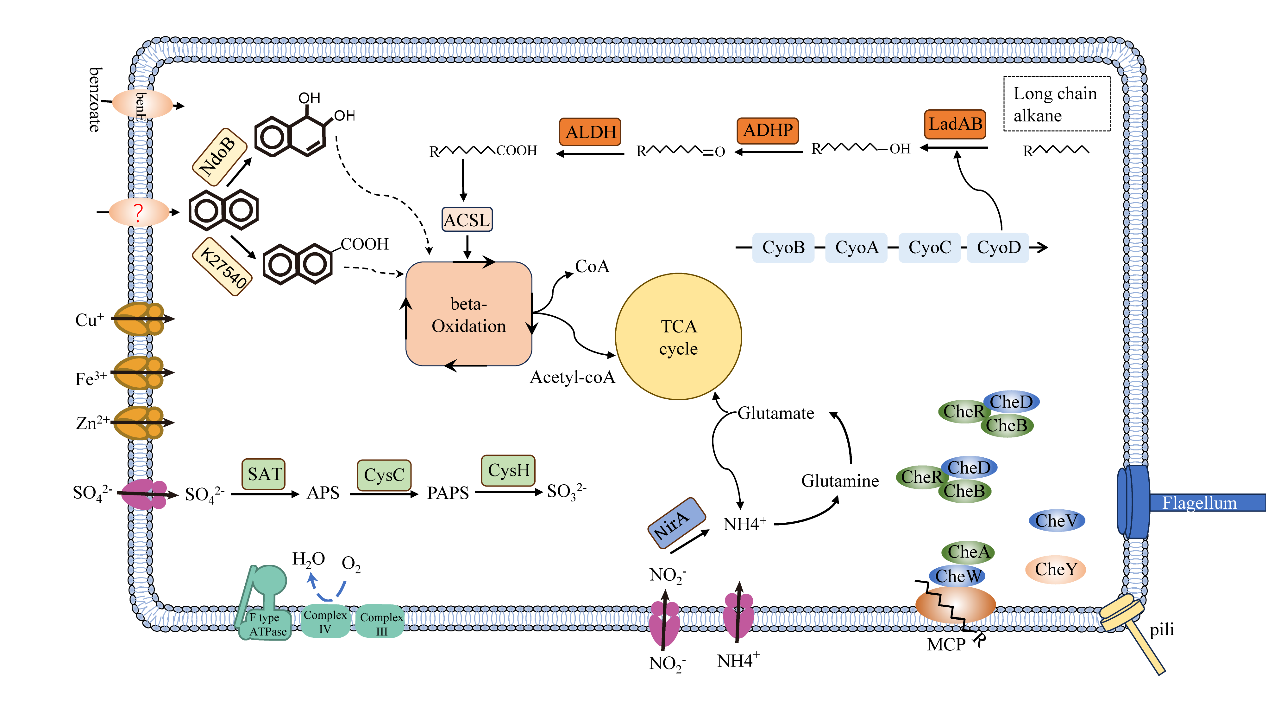


**Fig. S13 Putative hydrocarbon-degrading pathway of *Brevibacillus agri* (M539-542cm_MAG50) from the phylum** **Firmicutes.**


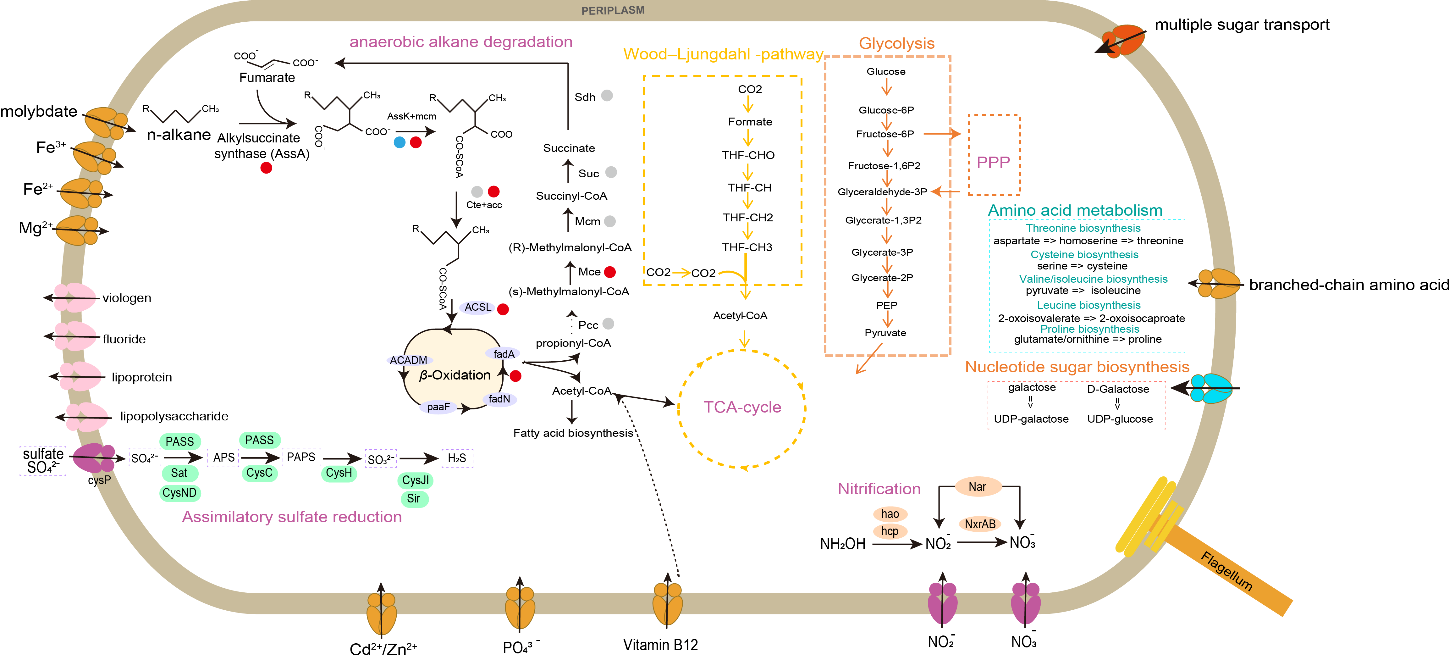


**Fig. S14 Proposed metabolic pathways for a novel anaerobic alkane-degrading MAG (M200-203cm_MAG22) from the phylum *Poribacteria* from the hadal sediment.**


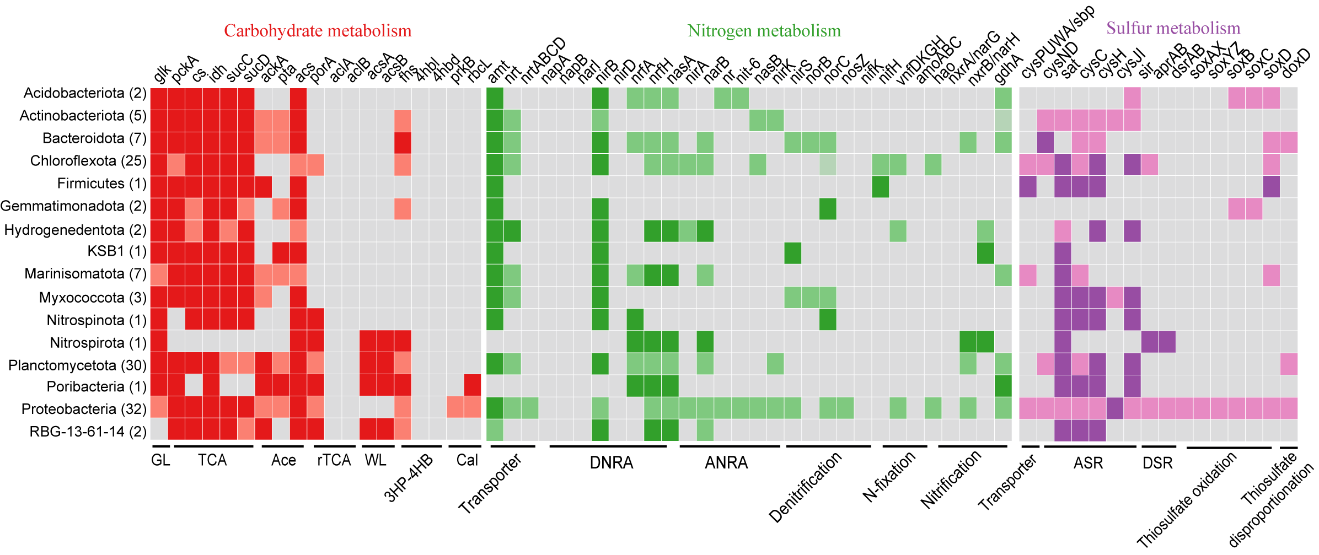


**Fig. S15 Heatmap of various putative metabolic genes across phyla found in 120 potential hydrocarbon-degrading MAGs.** Gl: glycolysis and gluconeogenesis, TCA: tricarboxylic acid cycle, rTCA: reductive tricarboxylic acid cycle, Ace: acetate metabolism, WL: Wood–Ljungdahl pathway, 3HP/4HB: 3-hydroxypropionate/4-hydroxybutyrate cycle, Cal: Calvin cycle, DNRA: Dissimilatory nitrate reduction to ammonia, ANRA: assimilatory nitrate reduction to ammonia, ASR: assimilatory sulfate reduction, DSR; dissimilatory sulfate reduction.


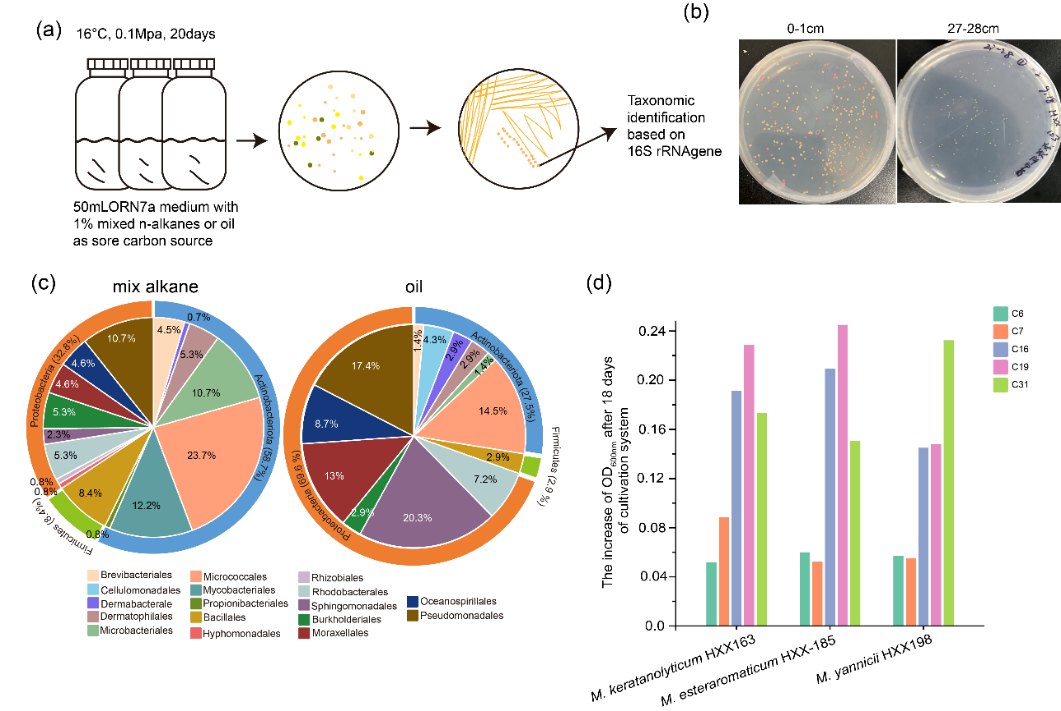


**Fig. S16 Isolation and functional verification of bacterial isolates capable of hydrocarbon degradation.** (a) Schematic detailing the enrichment and isolation method for potential hydrocarbon degrading bacteria from 23 sediment layers; (b) The visible bacterial colonies from two sediment samples (0-1cm and 27-28cm) cultured on mixed alkanes (C_6_, C_11_, C_16_, C_19_, C_22_, C_25_, C_28_ and C_32_) as the sole carbon source. (c) The community composition of cultivable hydrocarbon degrading bacteria on medium with mixed alkanes and petroleum. (d) Degradation ability of *n*-alkanes with different chain lengths for three isolates from the genus *Microbacterium*.


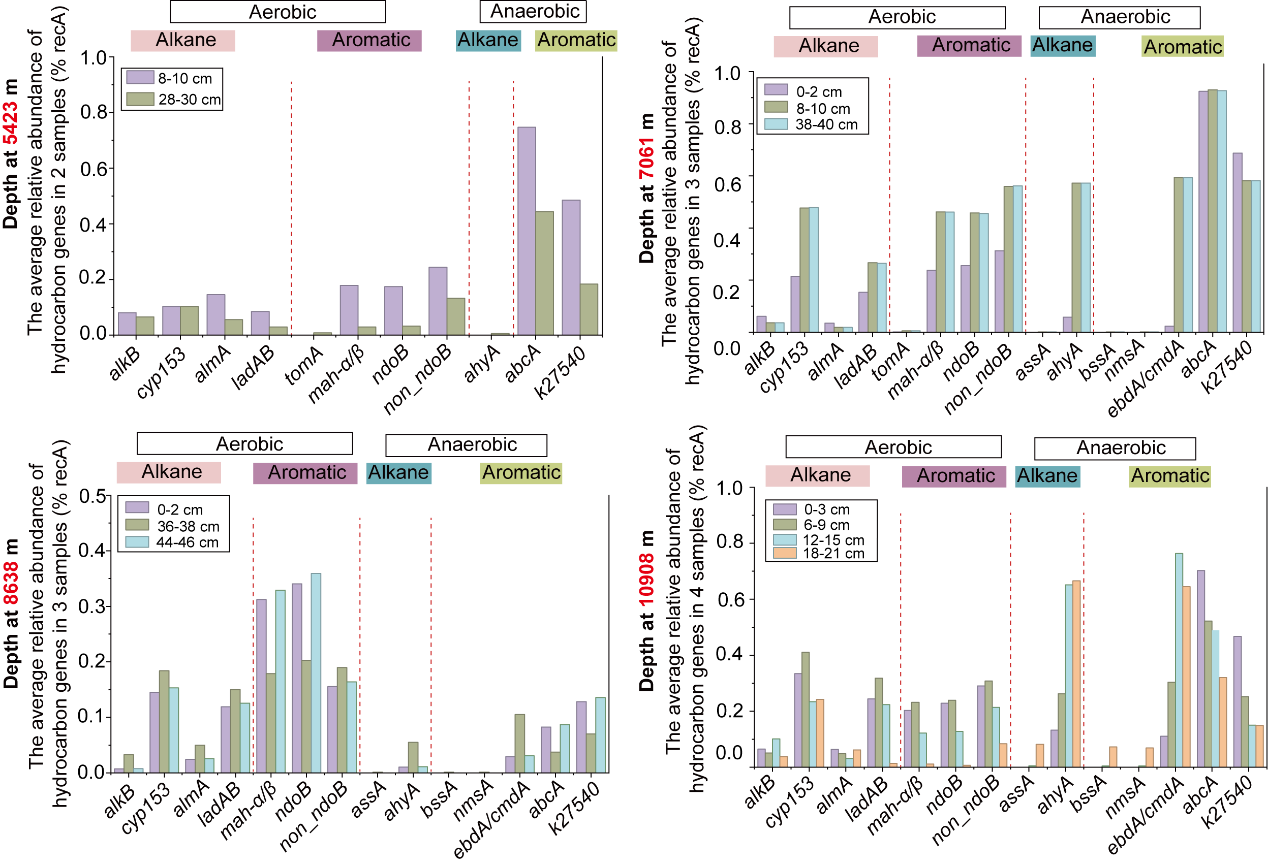


**Fig. S17 Relative abundance of HYD genes in four stations at different depths of the Mariana Trench.** The relative abundance of the diverse HYD genes across different depth layers in the Mariana Trench sediments at depth of 5423 m, 7061 m, 8683 m and 10908 m, respectively. The metagenome data used were retrieved from Zhou et al. (2022, Nature Communications, 13(1): 1515) (3).


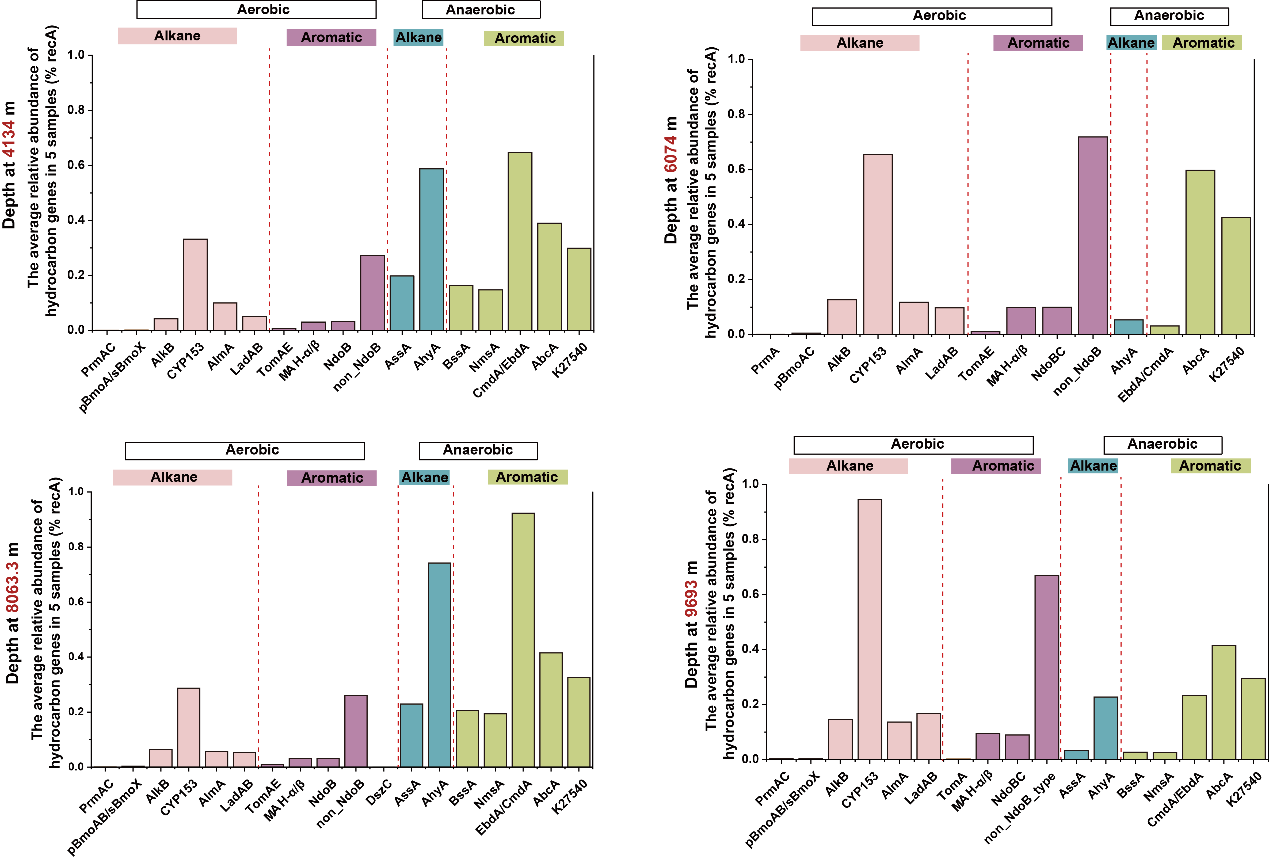


**Fig. S18 Relative abundance of diverse HYD genes in sediments from four stations at different depths (4143 m, 6074 m, 8063.3 m and 9693 m) in the Atacama Trench.** The metagenome data used were retrieved from Schauberger et al. (2024, ISME communications, 4(1): ycad005) (4).

**
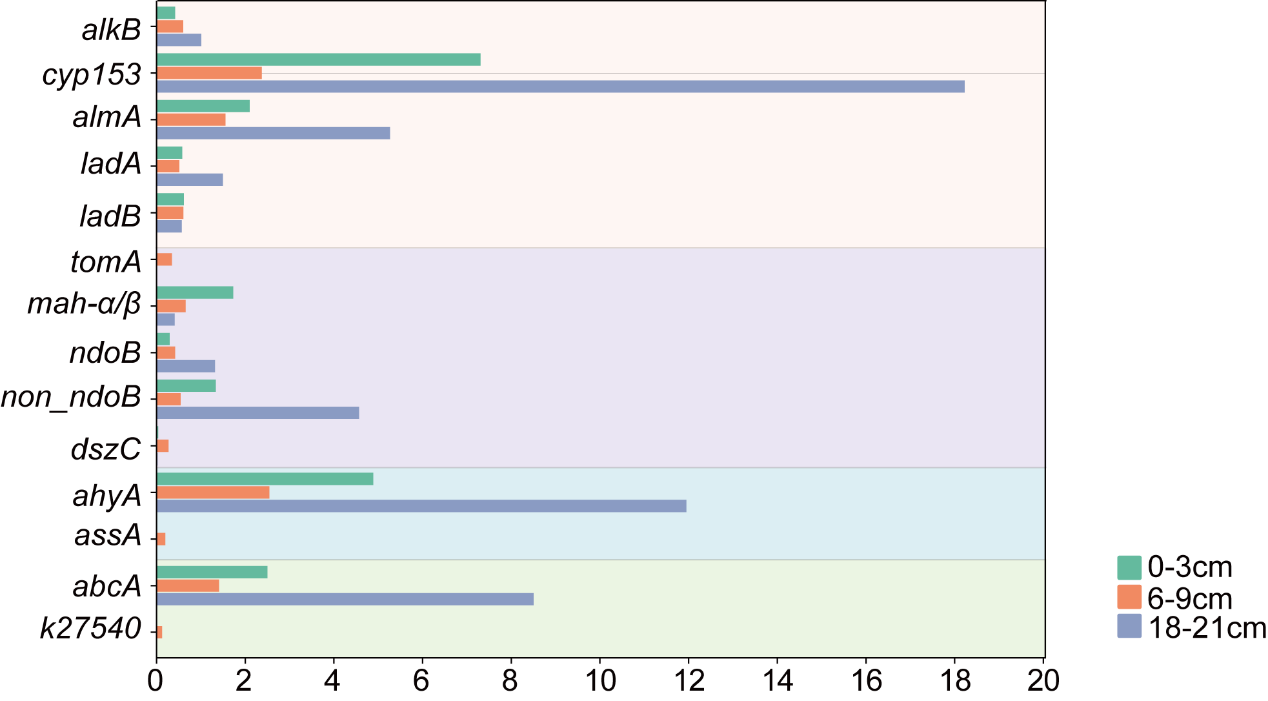
**

**Fig. S19 Transcriptional profiling of HYD genes (TPM) in the abyssal sediments of the Challenger Deep.** The expression levels of hydrocarbon degradation genes were examined by mapping publicly available metatranscriptomic reads (SRR12883926, SRR12883927 and SRR12883928, corresponding to three hadal sediment samples 6-9 cmbsf, 12-15 cmbsf, and 18-21 cmbsf at depths of 10,908 meters) Zhou et al. (2022, Nature Communications, 13(1): 1515) (3).

**References:**

(1) Liu J, Li D-W, He X, Liu R, Cheng H, Su C, Chen M, Wang Y, Zhao Z, Xu H, Cheng Z, Wang Z, Pedentchouk N, Lea-Smith DJ, Todd JD, Liu X, Zhao M, Zhang X-H. 2024. A unique subseafloor microbiosphere in the Mariana Trench driven by episodic sedimentation. Mar Life Sci & Technol 6:168-181.

(2) Wang W, Wang L, Shao Z. 2018. Polycyclic aromatic hydrocarbon (PAH) degradation pathways of the obligate marine PAH degrader *Cycloclasticus* sp. strain P1. Appl Environ Microb 84:e01261-18.

(3) Zhou Y-L, Mara P, Cui G-J, Edgcomb VP, Wang Y. 2022. Microbiomes in the Challenger Deep slope and bottom-axis sediments. Nat Commun 13:1515.

(4) Schauberger C, Thamdrup B, Lemonnier C, Trouche B, Poulain J, Wincker P, Arnaud-Haond S, Glud RN, Maignien L. 2024. Metagenome-assembled genomes of deep-sea sediments: changes in microbial functional potential lag behind redox transitions. ISME Commun. 4(1): ycad005.

**Supplementary file 2- Supplementary Tables**

**Table S1. *n*-alkane content in the sediment core.** The short-chain length *n*-alkanes (< C_16_) were not detected in the sediments, while medium-chain (C_18-24_) and long-chain length (C_25-36_) *n*-alkane were present in the core.

**Table S2. General features of the 22 metagenomic datasets**.

**Table S3. The 23 subsamples for microbial cultivation and formulation of ORN7a medium.**

**Table S4. Basic information on 2,400 hydrocarbon oxidation genes was predicted from 22 metagenomes.**

**Table S5. Summary of source information (at the phylum level) for 2,400 hydrocarbon oxidation genes predicted based on the NCBI-nr database and their relative abundances.**

**Table S6. Basic information of 342 dereplicated MAGs.**

**Table S7. Basic information on hydrocarbon-degrading related genes was predicted from 342 dereplicated MAGs.** Among these, 120 MAGs (~35.1%) were predicted to encode hydrocarbon-degrading related genes.

**Table S8. Various metabolic genes in carbon, nitrogen, and sulfur cycling across phyla identified in 120 potential hydrocarbon-degrading MAGs.**

**Table S9. Binding energies of AhyA Protein from Chloroflexota and two reference protein with alkanes of different chain lengths (C_10_ and C_20_) based on Autodock.**

**Table S10. Summary of studies reporting on potential hydrocarbon-degrading bacteria isolated from the sediment core MT20-750.**

**Table S11. Annotation of potential hydrocarbon synthesis-related genes in 342 dereplicate MAGs.**

**Table S12. Summary of hydrocarbon concentrations in coastal, abyssal, and hadal sediments.**

**Table S13. *n*-Alkane composition in the Challenger Deep sediment core MT20-750 and the overlying water.**
